# Use of a Retinal Phantom to Facilitate Adaptive Optics Scanning Light Ophthalmoscopy Imaging in a Multicenter Clinical Trial

**DOI:** 10.1167/tvst.15.8.20

**Published:** 2026-08-24

**Authors:** Joseph Kreis, Jessica Wong, Yushan Yang, Palash Bharadwaj, Robert F. Cooper, Mina Gaffney, Nivedhitha Govindasamy, Amy L. Green, Brian P. Higgins, Yu You Jiang, Angelos Kalitzeos, Michel Michaelides, Jessica I. W. Morgan, Jason Porter, Jeremy D. Rogers, Ian Rosenthal, Ramkumar Sabesan, Kaitlyn A. Sapoznik, Alexander W. Schill, Ryan D. Sochol, Yiyi Wang, Raymond L. Warner, Emma Warr, Benjamin J. Wendel, Niamh Wynne, Peiluo Xu, Sergey Tarima, Daniel X. Hammer, Anant Agrawal, Jacque L. Duncan, Joseph Carroll

**Affiliations:** 1 Department of Cell Biology, Neurobiology & Anatomy, Medical College of Wisconsin, Milwaukee, WI, USA; 2 Department of Ophthalmology, University of California, San Francisco, San Francisco, CA, USA; 3 Division of Biostatistics, Data Science Institute, Medical College of Wisconsin, Milwaukee, Wisconsin, USA; 4 Department of Ophthalmology, University of Washington School of Medicine, Seattle, WA, USA; 5 Roger and Angie Karalis Johnson Retina Center, Seattle, WA, USA; 6 Joint Department of Biomedical Engineering, Marquette University and Medical College of Wisconsin, Milwaukee, WI, USA; 7 Department of Ophthalmology & Visual Sciences, Medical College of Wisconsin, Milwaukee, WI, USA; 8 Bioengineering Graduate Group, University of Pennsylvania, Philadelphia, PA, USA; 9 UCL Institute of Ophthalmology, University College London, London, UK; 10 Moorfields Eye Hospital NHS Foundation Trust, London, UK; 11 Scheie Eye Institute, Department of Ophthalmology, Perelman School of Medicine, University of Pennsylvania, Philadelphia, PA, USA; 12 College of Optometry, University of Houston, Houston, TX, USA; 13 Department of Ophthalmology and Visual Sciences, University of Wisconsin-Madison, Madison, WI, USA; 14 McPherson Eye Research Institute, Madison, WI, USA; 15 Division of Biomedical Physics, Office of Science and Engineering Laboratories, Center for Devices and Radiological Health, US Food and Drug Administration, Silver Spring, MD, USA; 16 Department of Mechanical Engineering, University of Maryland, College Park, MD, USA; 17 Southern California College of Optometry, Marshall B. Ketchum University, Fullerton, CA, USA; 18 Department of Biostatistics, University of Kentucky, Lexington, KY, USA

**Keywords:** adaptive optics, retinal phantom, clinical trials, reproducibility, retinal imaging

## Abstract

**Purpose:**

To describe the use of a model eye-mounted retinal phantom to support adaptive optics scanning light ophthalmoscope (AOSLO) imaging in a multicenter clinical trial (NCT05537220).

**Methods:**

Six three-dimensional–printed retinal phantoms were mounted in model eyes. These phantoms contain equally spaced arrays of reflective “cone-like” structures mimicking foveal cone photoreceptor spacing. Intrasession repeatability of phantom imaging was assessed for all six phantoms on two custom AOSLOs. One phantom was also imaged on two additional AOSLOs to assess interdevice reproducibility. Lastly, each of the six phantoms was imaged longitudinally at their respective trial sites.

**Results:**

Intrasession repeatability of phantom-derived horizontal “cone” spacing measures was high for two different AOSLOs. However, intersystem differences were found in the average horizontal “cone” spacing of five of eight patches assessed. Across four unique AOSLOs, intersystem differences were found in the average horizontal “cone” spacing of seven of eight patches assessed. A linear regression analysis of longitudinal phantom imaging with a single AOSLO revealed few significant changes in horizontal “cone” spacing over the course of about 2 years. Similar results were observed for vertical “cone” spacing measures across the three experiments.

**Conclusions:**

Longitudinal spacing measurements were generally stable in our study. However, small but significant differences in spacing were observed between systems. The approach described here may facilitate combining cone metrics extracted from images acquired using different AOSLOs in multicenter studies.

**Translational Relevance:**

Cross-system validation strategies can support the use of AOSLO imaging in multicenter clinical trials.

## Introduction

Adaptive optics scanning light ophthalmoscope (AOSLO) imaging enables noninvasive, cellular-resolution visualization of the living human retina.^1^ As such, AOSLO imaging has been demonstrated as a sensitive tool for detecting changes in retinal pathology, enabling accurate assessment of disease progression and improved evaluation of response to therapeutic intervention.^2^ Since its inception in the early 2000s, AOSLO imaging has been utilized in hundreds of studies to examine the pathophysiology of retinal and systemic diseases.^3^^,^^4^ Clinical applications of AOSLO imaging have largely involved studies of conditions affecting the cone photoreceptors due both to our reliance on cone-mediated vision and the ease with which cones can be visualized.^5^^–^^7^ Additional studies have included adaptive optics (AO)–based functional assessments (including visual acuity, microperimetry, and optoretinography), which have provided mechanistic structure–function insights across various retinal conditions.^8^^–^^18^

Despite these advances, the use of AOSLO imaging in clinical trials has been relatively limited (Jiang YY, et al. *IOVS* 2024;65:ARVO E-Abstract 3289).^19^^–^^32^ In one study, Talcott et al.^21^ described AOSLO-derived cone spacing and density metrics for three patients with retinitis pigmentosa (RP) in a phase 2 trial.^20^ They found more stable cone metrics in eyes receiving ciliary neurotrophic factor (CNTF) treatment compared to contralateral eyes receiving sham treatment, suggesting a possible therapeutic effect at a cellular level. This work was continued with a subsequent trial assessing the cone mosaic of 22 patients with RP at baseline and 36 months after CNTF treatment.^22^ In a natural history study of 51 patients with *CNGB3*-associated achromatopsia (ACHM), Langlo et al.^6,27^ confirmed the presence of residual foveal cone structure despite a dark cone phenotype on confocal AOSLO imaging, and follow-up imaging of 41 of these patients with ACHM revealed stable foveal cone structure over a span of up to 26 months.^24^ It is worth noting that while patients originated from multiple clinical sites in these ACHM studies, they traveled to a single site to complete AOSLO imaging (an expensive and time-consuming endeavor). Additional trials using AOSLO imaging have included the assessment of cone density changes following treatment for macular telangiectasia type 2^30^ and measurement of changes in blood flow after treatment for multiple sclerosis.^31^ Further studies are also recruiting patients with RP, choroideremia, age-related macular degeneration, and other conditions to develop novel retinal biomarkers of disease and promote the clinical use of AOSLO imaging in natural history studies of disease progression.^19^^,^^23^^,^^29^^,^^32^

Even rarer have been multicenter trials involving AOSLO imaging. At least three studies have followed patients longitudinally over a span of up to 2 years. In a multicenter phase 2 trial evaluating implants for CNTF delivery into the eyes of patients with macular telangiectasia type 2, AOSLO-derived changes in cone density were included as a secondary outcome measure, although the AOSLO-based results of this study have not been published.^25^ Most recently, a natural history study from Duncan et al.^33^ reported an increase in cone spacing over 24 months in patients with *USH2A*-related degeneration who completed AOSLO imaging across four sites. This study emphasized the value of additional multisite trials to enhance and refine quantitative analyses of cone structure and develop reliable therapeutic outcome measurements with data acquired across multiple unique AOSLOs. At least one study has begun addressing these needs—the “Oral N-acetylcysteine for Retinitis Pigmentosa (NAC Attack)” multicenter, phase 3 clinical trial (NCT05537220) includes a six-site AOSLO imaging substudy to assess cone photoreceptor structure in patients with RP who are taking N-acetylcysteine (NAC) or placebo for 45 months.^34^^,^^35^

One reason for the limited use of AOSLO imaging in multicenter trials is nonuniformity in investigational device design. As a variety of features, including image resolution and scale, can vary as a function of system design, comparing and combining data acquired on different systems can be challenging. One cross-system validation strategy involves the use of retinal phantoms, an approach that facilitated the development and quick clinical adaptation of optical coherence tomography (OCT) in the 2000s.^36^ Phantoms are biomimetic tools that are valuable for the development of novel imaging modalities and for assessing device performance. In ocular imaging, phantoms have been developed as an imaging standard with known optical properties that can mimic the human eye.^37^^–^^39^ This has enabled repeatable methods of validating ophthalmic devices, comparing different instruments, and ensuring high accuracy and quality in ocular imaging.^38^^,^^40^^–^^42^ Retinal phantoms are now being developed for adaptive optics imaging systems,^42^^,^^43^ fabricated to mimic the reflectivity and spacing of parafoveal cone photoreceptors.^44^ Here we sought to evaluate the use of a model eye-mounted retinal phantom to facilitate the integration of AOSLO-derived cone mosaic metrics from images acquired across six unique systems in the AOSLO imaging substudy of the NAC Attack multicenter, phase 3 clinical trial.^34^^,^^35^

## Methods

### AOSLO Model Eye-Mounted Retinal Phantom

**Figure 1.**
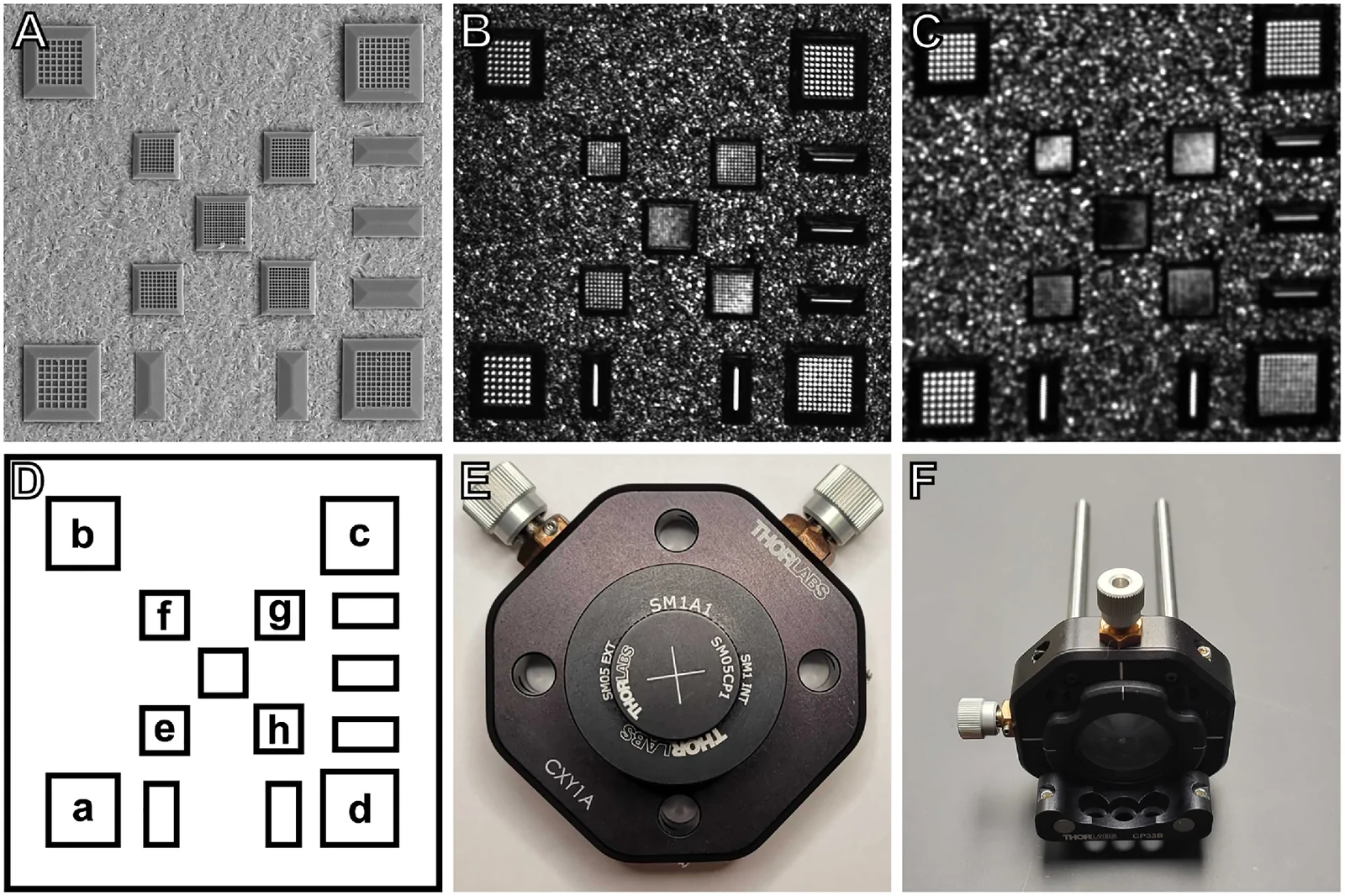
A model eye-mounted retinal phantom for AOSLO imaging. (**A**) Electron microscopy imaging shows a 3D-printed retinal phantom, which comprises nine patches of “cone-like” reflective structures, each with a different spacing mimicking the human foveal and parafoveal cone mosaic. Reflected light microscopy of a single retinal phantom (not shown) was used to validate the known spacing of each patch's “cones” after fabrication. The known and vertical “cone” spacings are listed in Table 2 and Supplementary Table S2. (**B**) The retinal phantoms were imaged with bespoke AOSLOs, which are being used to image human research participants enrolled in a clinical trial. This image is of the retinal phantom used for monitoring AOSLO 1. (**C**) The same retinal phantom was also imaged on a newly available commercial AOSLO (Mona IV; Robotrak Technologies, Nanjing, China) for qualitative comparison to images from our bespoke AOSLOs. (**D**) A mask of the retinal phantoms’ patches is labeled as they are referred to in the text and subsequent figures and tables. “Horizontal” refers to measurements in the a→d direction, and “vertical” refers to measurements in the c→d direction, regardless of the orientation of a phantom relative to the AOSLO used to image it. (**E**) The retinal phantoms were mounted in off-the-shelf optomechanical components to facilitate AOSLO imaging. (**F**) The housing of the retinal phantom was placed on rails to insert the model eye into each AOSLO at the same plane as a standard model eye would be imaged.

Six model eyes were constructed in March 2022, each incorporating a three-dimensional (3D)–printed retinal phantom printed with Nanoscribe (Fig. 1).^44^ Each model eye was constructed using an achromatic doublet lens (49-952-INK; Edmund Optics, Barrington, NJ, USA) with a focal length of 17.5 mm and a diameter of 12.5 mm, modeling the refraction of an emmetropic human eye. The lens has an antireflective coating for visible and near-infrared imaging with an inked edge. The model eye housing was constructed using off-the-shelf optomechanical components (ThorLabs, Newton, NJ, USA) (Figs. 1E, 1F). The posterior chamber depth of each model eye-mounted retinal phantom was verified using the process described by Agrawal and colleagues^44^ to minimize the sphere (defocus) term recorded by a wavefront sensor. The retinal phantom, mounted within the model eye, comprises nine square patches, each containing an equally spaced array of reflective structures mimicking the cellular structure of cone photoreceptors (Fig. 1A). Each patch emulates cone spacing at a different retinal eccentricity, with center-to-center spacing of the cone-like structures ranging from 1.92 µm (the center patch) to 4.97 µm. The retinal phantom also includes a set of horizontal and vertical bars on two of its sides, which may be used for measuring the X-Y scale of the system. The “cone” spacing of each patch on a seventh retinal phantom printed in the same batch was validated with reflected light microscopy.

### AOSLO Imaging of the Retinal Phantom

Images of the retinal phantom (Fig. 1B) were acquired on six bespoke AOSLOs. In our first experiment to assess intrasession repeatability, each retinal phantom was imaged twice within a single imaging session on two different days using AOSLO 1 and AOSLO 2: the Medical College of Wisconsin^45^^,^^46^ and the University of California, San Francisco, respectively.^47^ A follow-up experiment was completed wherein one of the retinal phantoms was also imaged once using AOSLO 7 and AOSLO 8: the University of Wisconsin, Madison^48^ and Marquette University, respectively.^49^ In a third experiment, the retinal phantoms were imaged over the span of approximately 2 years at the six sites involved in the NAC Attack AOSLO imaging substudy (AOSLOs 1–6): the Medical College of Wisconsin; the University of California, San Francisco; the University of Washington^50^; the University of Houston^51^; Moorfields Eye Hospital/University College London^48^; and the University of Pennsylvania, respectively.^45^^,^^46^

For imaging the retinal phantom, AOSLO operators were instructed to place the lens of the model eye at the same pupil plane where a standard model eye or calibration grid would be placed in each AOSLO. Two adjustment knobs on the model eye were used to translate the retinal phantom and center it within the imaging window horizontally and vertically (Figs. 1E, 1F). Once the retinal phantom was centered, the AOSLO's system focus was adjusted to optimize the resolution of the cone-like structures in the confocal detection channel, and the control voltage of the confocal detector was adjusted to ensure the image was not saturated. Imaging sequences (number of frames varied by site) were acquired using a 1 × 1° field of view (FOV) and were processed by personnel at each site to generate desinusoided, registered, and averaged images for analysis. While the specific processing pipeline varied slightly between sites, each generally followed previously described methods.^52^^,^^53^

### “Cone” Spacing Assessment

The horizontal and vertical scale of each image in microns per pixel was determined using information from calibration grids imaged the same day as the retinal phantom. This was calculated as the phantom model eye's estimated scale on a retinal plane of 305.5 µm/deg (given tan(1°) × 17.5 mm), multiplied by the true scan angle of the system (equal to or approximately 1°) and divided by the desinusoided image size in pixels. Using the “Straight Line” tool in ImageJ,^54^ a pixel intensity plot profile was generated for both a central row and column of cone-like features in eight of the nine patches of the phantom image (Supplementary Fig. S1). The central patch of the phantom was not assessed as its “cone” spacing is below the resolution limit for some of the AOSLOs used in this study, which was estimated to be between 1.80 and 1.91 µm, given their imaging wavelengths of 790 nm and 840 nm, respectively. A peak-to-peak measurement was made to determine the pixel spacing of the “cones” in each of the eight patches analyzed in each retinal phantom image (Supplementary Fig. S1). This measurement was multiplied by the previously calculated microns per pixel value to convert the “cone” spacing from pixels to microns for each individual image. We report horizontal “cone” spacing here as measurements made along the orientation of patch “a” to patch “d” (as labeled in Fig. 1D). Corresponding vertical “cone” spacing measurements, or those along the orientation of patch “c” to patch “d,” as labeled in Figure 1D, are reported in supplementary figures and tables. Analyses were completed according to the measurement orientation regardless of the scan direction a measurement was made with or against.

### Statistical Analyses

Unless otherwise specified, all statistical calculations were completed using RStudio v4.5.1. All images included in the analyses presented are provided in Supplementary Material S1, and the code used for statistical analyses can be shared upon request. All statistical analyses were exploratory and relied on linear and linear mixed methods, including random-effect models and simple linear regressions. Bland–Altman plots and limits of agreement were used for repeatability analyses. Variance-stabilizing transformations were used when Shapiro–Wilk tests determined nonnormality of distributions.

### Measurement Error

Prior to testing the intrasession repeatability and interdevice reproducibility of measured “cone” spacing for the eight patches on the retinal phantoms imaged with an AOSLO, we calculated an overall measurement error^55^ for the single observer (JK) following their two repeat measurements of the 52 images across Experiment 1 (48 images, intra- and intersession repeatability) and Experiment 2 (four images, interdevice reproducibility) in both horizontal and vertical dimensions. The repeated measures and the calculation of measurement error are presented in Supplementary Material S2. The distribution of measurements was nonnormal (*P* < 0.0001, Shapiro–Wilk), so a logarithmic transformation (base *e*) was used to estimate the measurement error of the single observer.^56^ The measurement error (coefficient of variation) was 2.19% (or 0.026 µm on a logarithmic scale). Due to the small measurement error, any repeated measures were averaged for an image before completing the statistical tests for Experiment 1 and Experiment 2.

### Experiment 1: Intrasession and Intersession Repeatability

All six model eye-mounted retinal phantoms were imaged in two sessions on each of two different days with AOSLO 1.^45^^,^^46^ In each session, each retinal phantom was imaged twice at a 1 × 1° FOV, resulting in 24 total images. With eight patches assessed on each retinal phantom image (Fig. 1D), the repeated imaging resulted in 192 total patches assessed. The patches on each retinal phantom image were measured twice in both the horizontal and vertical dimensions for a total of 768 measurements for AOSLO 1. Imaging the retinal phantoms occurred in the same order for both sessions, and assessments were made for each retinal phantom in the same order they were imaged. This imaging and assessment scheme was then repeated on AOSLO 2 for another 768 measurements. While the AOSLO and imaging personnel differed, all assessments of images acquired on AOSLO 2 were made by the same observer, who assessed the images acquired on AOSLO 1 (JK).

To assess the intrasession repeatability of placing the retinal phantom into the AOSLO for repeated imaging, Bland–Altman analyses were completed for each of the four imaging sessions across the two AOSLOs.^64^ This included estimating the mean bias, the 95% limits of agreement, and the 99% confidence intervals (CIs) around the mean bias and limits of agreement, which were calculated for the average of the repeated measurements for each round of images acquired within each imaging session. A linear mixed-effects model was used to further explore differences between the two images acquired within each session as well as between imaging sessions or between the AOSLOs (all considered fixed effects). The model was fitted using the method of restricted maximum likelihood (separately for each predetermined patch spacing), and the retinal phantom was included as a random effect to determine the contribution of the retinal phantoms to the variance of repeated measurements. The random effect of the retinal phantom was assumed to be normally distributed with a mean of zero. A separate series of linear mixed-effects models was also used to assess whether any individual retinal phantom (fixed effect) deviated from the other retinal phantoms in the spacing measurements, where imaging session and image were treated as random effects.

### Experiment 2: Interdevice Reproducibility

One of the six model eye-mounted retinal phantoms was imaged once more on both AOSLO 7 and AOSLO 8 to examine interdevice differences. In each imaging session, the phantom was imaged once at a nominal 1 × 1° FOV. With eight patches assessed on each phantom image, these two images and a representative image from Experiment 1 for both AOSLO 1 and AOSLO 2 resulted in four images with 32 total patches assessed. Measurements occurred in numerical order of AOSLO by a single observer (JK).

A linear mixed-effects model was used with system as a fixed effect and image as a random effect to assess the difference between measured and known “cone” spacing values for each patch on the retinal phantom across the four AOSLOs used for imaging in this experiment. Similar to the final model from Experiment 1, another separate linear mixed-effects model was also used to assess whether any AOSLO systematically differed from the other three for each patch, where AOSLO was treated as a fixed effect and image was treated as a random effect.

### Experiment 3: Longitudinal Changes in “Cone” Spacing

Each model eye-mounted retinal phantom was shipped to one of the six sites in the NAC Attack AOSLO substudy (AOSLOs 1–6), where they are being used to monitor each site's AOSLO longitudinally during the NAC Attack clinical trial. All retinal phantom images submitted for this study were measured once by a single observer (JK). The number of imaging sessions varied per site, as did the time between the first session and the final session included in this analysis (Supplementary Fig. S2).

For each combination of AOSLO and retinal phantom image patch, a simple linear regression model was fit with time (i.e., days since baseline) as the predictor and difference from the known “cone” spacing (in microns) as the response.

## Results

### Experiment 1: Intrasession and Intersession Repeatability

**Figure 2.**
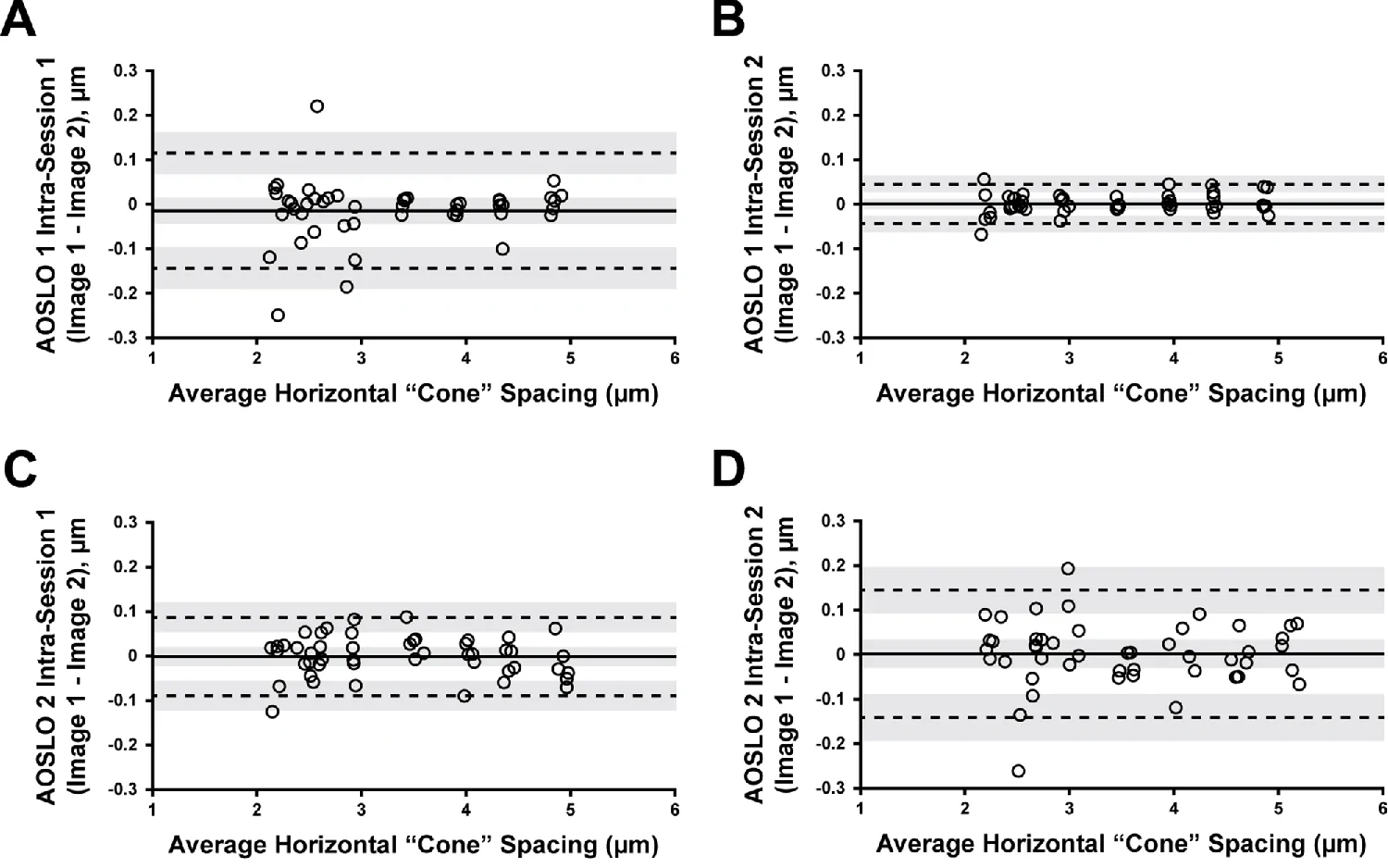
Intrasession agreement of horizontal “cone” spacing for six retinal phantoms. (**A**) We observed strong agreement of horizontal “cone” spacing between two sets of images acquired in a single session on AOSLO 1. The *solid line* represents the mean bias of −0.014 µm, and the *dashed lines* represent the upper (0.115 µm) and lower (−0.144 µm) limits of agreement. (**B**) Strong agreement was also observed for horizontal “cone” spacing between two sets of images acquired during a separate imaging session on AOSLO 1. The *solid line* represents the mean bias of 0.001 µm, and the *dashed lines* represent the upper (0.046 µm) and lower (−0.043 µm) limits of agreement. (**C**) We observed strong agreement on AOSLO 2 for horizontal “cone” spacing between repeated imaging within the same session as well. The *solid line* represents the mean bias of −0.002 µm, and the *dashed lines* represent the upper (0.087 µm) and lower (−0.090 µm) limits of agreement. (**D**) Strong agreement was also observed for horizontal “cone” spacing between repeated imaging in a second session on another day on AOSLO 2. The *solid line* represents the mean bias of 0.002 µm, and the *dashed lines* represent the upper (0.145 µm) and lower (−0.141 µm) limits of agreement. The *gray shading* represents the 99% CIs about the mean bias and the limits of agreement.^64^ Each vertical grouping of data points indicates a patch on the phantom, with patch “a” being the greatest spacing on the right and patch “h” being the smallest spacing on the left. Similar results were observed for vertical “cone” spacing measures (Supplementary Fig. S3).

Bland–Altman analysis of horizontal “cone” spacing measurements showed strong agreement of phantom imaging across four imaging sessions of all six retinal phantoms on AOSLO 1 and AOSLO 2. For all four imaging sessions, the 99% CIs of the mean difference included zero, suggesting there was no significant difference between images when imaging the six retinal phantoms twice in the same imaging session on either AOSLO 1 or AOSLO 2 (Fig. 2). Bland–Altman analysis of vertical “cone” spacing measurements revealed similar results (Supplementary Fig. S3).

Another linear mixed-effects model confirmed that there were no significant differences between same-session images for horizontal “cone” spacing (*P* values between 0.2797 for patch “h” and 0.9927 for patch “e”). There was, however, one patch with a significant difference between same-session images for vertical “cone” spacing (“b”; *P* = 0.0297). Despite no significant intrasession differences in horizontal “cone” spacing, patch-dependent session effects were found such that patches “a,” “b,” “c,” “d,” “e,” “g,” and “h” all had significant differences between sessions (*P* values between 0.0000 and 0.0464) while patch “f” did not (*P* = 0.2257). For vertical “cone” spacing, patches “a,” “b,” “c,” “e,” “g,” and “h” all had significant differences between sessions (*P* values between 0.0000 and 0.0178), while patches “d” and “f” were not significantly different between sessions (*P* = 0.1240 and *P* = 0.3829, respectively). Further, the comparison of AOSLO 1 to AOSLO 2 revealed that “cone” spacing measurements from images acquired with AOSLO 2 were significantly greater for seven of the eight patches for both horizontal and vertical “cone” spacing (all *P* values <0.0098).

While we did not find any significant intrasession differences overall for repeated imaging of the retinal phantoms on either AOSLO, we did find more subtle differences in “cone” spacing between imaging session and AOSLO. This linear mixed-effects model also enabled the assessment of retinal phantom contribution (random effect) to the variability of the differences of the measured “cone” spacing from the known “cone” spacings. Across the eight patches on the retinal phantom, between 0% (patch “d”) and 21.05% (patch “c”) of the variance in “cone” spacing measurements could be explained by differences between the six retinal phantoms, suggesting that residual variance (i.e., something other than differences between phantoms) is the most prevalent source of variance across measurements.

To directly assess whether any individual retinal phantom's horizontal “cone” spacing measurements were systematically different from the average of the other five retinal phantoms, we also ran a linear mixed-effects model. This analysis revealed that four of the six retinal phantoms had at least one patch with a horizontal “cone” spacing significantly different from the average “cone” spacing of the same patch on the other five retinal phantoms (Table 1). The greatest difference found across the six retinal phantoms was for patch “c” on retinal phantom 5, which had a horizontal “cone” spacing 0.050 µm greater than the other phantoms (*P* = 0.0024). The smallest difference found to be significant was the horizontal “cone” spacing for patch “c” on retinal phantom 6, which was 0.037 µm greater than on the other phantoms (*P* = 0.0259). Patches “d,” “f,” and “g” were not found to significantly differ between any of the retinal phantoms. Similar linear mixed-effects model results were found for vertical “cone” spacing, with the greatest significant difference found for patch “f” on retinal phantom 3 (0.101 µm greater; *P* = 0.0000) and the smallest significant difference found for patch “c” on retinal phantom 3 (0.026 µm greater; *P* = 0.0344) (Supplementary Table S1).

**Table 1.** Examining Differences in Horizontal “Cone” Spacing Measurements Between Phantoms

	Retinal Phantom 1		Retinal Phantom 2		Retinal Phantom 3		Retinal Phantom 4		Retinal Phantom 5		Retinal Phantom 6	
Patch	Effect Size^a^	*P* Value	Effect Size^a^	*P* Value	Effect Size^a^	*P* Value	Effect Size^a^	*P* Value	Effect Size^a^	*P* Value	Effect Size^a^	*P* Value
a	−0.0119	0.5087	−0.0305	0.0869	−0.0266	0.1345	0.0330	0.0630	−0.0029	0.8699	**0.0389**	**0.0280**
b	−0.0150	0.3712	−0.0085	0.6133	−0.0202	0.2281	−0.0227	0.1761	0.0203	0.2256	**0.0461**	**0.0052**
c	−**0.0421**	**0.0104**	−0.0295	0.0755	−0.0259	0.1187	0.0112	0.5040	**0.0496**	**0.0024**	**0.0368**	**0.0259**
d	−0.0196	0.1266	−0.0037	0.7707	0.0019	0.8802	0.0204	0.1113	−0.0026	0.8375	0.0036	0.7777
e	0.0149	0.4697	0.0085	0.6801	−0.0337	0.0992	−0.0344	0.0925	**0.0415**	**0.0417**	0.0032	0.8748
f	0.0328	0.0870	0.0071	0.7148	−0.0136	0.4820	−0.0095	0.6210	0.0112	0.5608	−0.0280	0.1453
g	−0.0414	0.0853	0.0193	0.4251	−0.0451	0.0605	−0.0023	0.9266	0.0403	0.0941	0.0292	0.2282
h	0.0169	0.3477	0.0019	0.9247	−**0.0442**	**0.0132**	0.0205	0.2558	0.0102	0.5726	−0.0050	0.7786

a Effect size provided in micrometers. Effect size = estimate of “cone” spacing difference between one phantom and an average of the other five. Bold = significant *P* value.

### Experiment 2: Interdevice Reproducibility

After one of the retinal phantoms was also imaged on AOSLO 7 and AOSLO 8, we sought to compare the AOSLOs via the horizontal “cone” spacing of each of the eight patches on the retinal phantom. Two measurements of a single 1 × 1° FOV image acquired on each of the four AOSLOs (1, 2, 7, and 8) were averaged to get a horizontal “cone” spacing measurement for each patch on the retinal phantom for each AOSLO. The known horizontal “cone” spacing, the measured horizontal “cone” spacing, the difference in microns between the known and measured horizontal “cone” spacing, and the difference as a percentage between the known and measured horizontal “cone” spacing for all eight patches on all four AOSLOs are summarized in Table 2. We observed that the measured horizontal spacing of each patch varies but is still close to the known horizontal spacing across the four AOSLOs (no greater than a 0.146-µm difference for any patch across the AOSLOs). Similar trends were observed for vertical “cone” spacing (Supplementary Table S2).

**Table 2.** Measured Versus Known Horizontal “Cone” Spacing Across Four AOSLOs

	a	b	c	d	e	f	g	h
Known spacing^a^	4.95	4.48	3.99	3.49	2.92	2.58	2.44	2.20
AOSLO 1								
Measured spacing^a^	4.89	4.39	3.96	3.47	2.93	2.58	2.49	2.12
Difference (µm)	−0.055	−0.087	−0.033	−0.024	0.012	−0.001	0.049	−0.082
Difference (%)	−1.12	−1.96	−0.84	−0.70	0.41	−0.05	1.99	−3.78
AOSLO 2								
Measured spacing^a^	4.94	4.45	4.09	3.59	2.91	2.61	2.39	2.15
Difference (µm)	−0.014	−0.034	0.105	0.100	−0.007	0.028	−0.048	−0.053
Difference (%)	−0.28	−0.76	2.59	2.81	−0.22	1.10	−1.99	−2.44
AOSLO 7								
Measured spacing^a^	4.97	4.46	3.90	3.43	2.93	2.59	2.44	2.16
Difference (µm)	0.025	−0.020	−0.093	−0.061	0.006	0.008	0.001	−0.038
Difference (%)	0.50	−0.44	−2.36	−1.76	0.20	0.32	0.05	−1.74
AOSLO 8								
Measured spacing^a^	5.10	4.56	4.09	3.58	2.94	2.67	2.54	2.29
Difference (µm)	0.146	0.076	0.097	0.086	0.023	0.094	0.099	0.085
Difference (%)	2.90	1.67	2.41	2.44	0.78	3.57	4.00	3.79
Four-system average								
Measured spacing^a^	4.98	4.46	4.01	3.52	2.93	2.61	2.47	2.18
Difference (µm)	0.025	−0.016	0.019	0.025	0.009	0.032	0.025	−0.022
Difference (%)	0.50	−0.37	0.45	0.70	0.29	1.23	1.01	−1.04

a Known and measured spacing values are in micrometers.

We also sought to assess whether any of the four AOSLOs systematically differed from the other three in their “cone” spacing measurements for the eight patches. The linear mixed-effects model analysis (Table 3) revealed that AOSLO 1 did not significantly differ from the other AOSLOs in horizontal “cone” spacing, and AOSLO 2 significantly differed from the other AOSLOs in the horizontal “cone” spacing of only patch “g” (0.10 µm smaller; *P* = 0.0333). AOSLO 7 and AOSLO 8 had two and five patches, respectively, that were significantly different from the other three AOSLOs, although the trends were opposite to each other. The two significant effects for AOSLO 7 were horizontal “cone” spacings that were less than the other AOSLOs, while the five significant effects for AOSLO 8 were all horizontal “cone” spacings that were greater than the other AOSLOs. Linear mixed-effects model results for vertical “cone” spacing are presented in Supplementary Table S3. It is worth noting that, given the number of comparisons made across these two linear mixed-effects models (96 total) and use of a standard α of 0.05, the probability of finding at least one significant result by chance was ∼99%.

**Table 3.** Assessing the Impact of AOSLO on Horizontal “Cone” Spacing Measurements

	AOSLO 1		AOSLO 2		AOSLO 7		AOSLO 8	
Patch	Effect Size^a^	*P* Value	Effect Size^a^	*P* Value	Effect Size^a^	*P* Value	Effect Size^a^	*P* Value
a	−0.1072	0.1097	−0.0524	0.474	−0.0008	0.9916	**0.1604**	**0.0017**
b	−0.0944	0.0594	−0.0233	0.6873	−0.0048	0.9343	**0.1225**	**0.0028**
c	−0.0696	0.3931	0.1144	0.1341	−**0.1493**	**0.0307**	0.1047	0.1784
d	−0.0661	0.3129	0.0993	0.1038	−**0.1148**	**0.0477**	0.0816	0.2011
e	0.0045	0.7336	−0.0200	0.0902	−0.0036	0.7872	0.0191	0.1112
f	−0.0448	0.3189	−0.0051	0.9145	−0.0320	0.4857	**0.0819**	**0.0353**
g	0.0315	0.5671	−**0.0979**	**0.0333**	−0.0324	0.5558	**0.0988**	**0.0308**
h	−0.0796	0.1694	−0.0415	0.5012	−0.0215	0.7315	**0.1427**	**0.0001**

a Effect size provided in micrometers. Effect size = estimate of “cone” spacing difference between one AOSLO and an average of the other three. Bold = significant *P* value.

### Experiment 3: Longitudinal Changes in “Cone” Spacing

**Figure 3.**
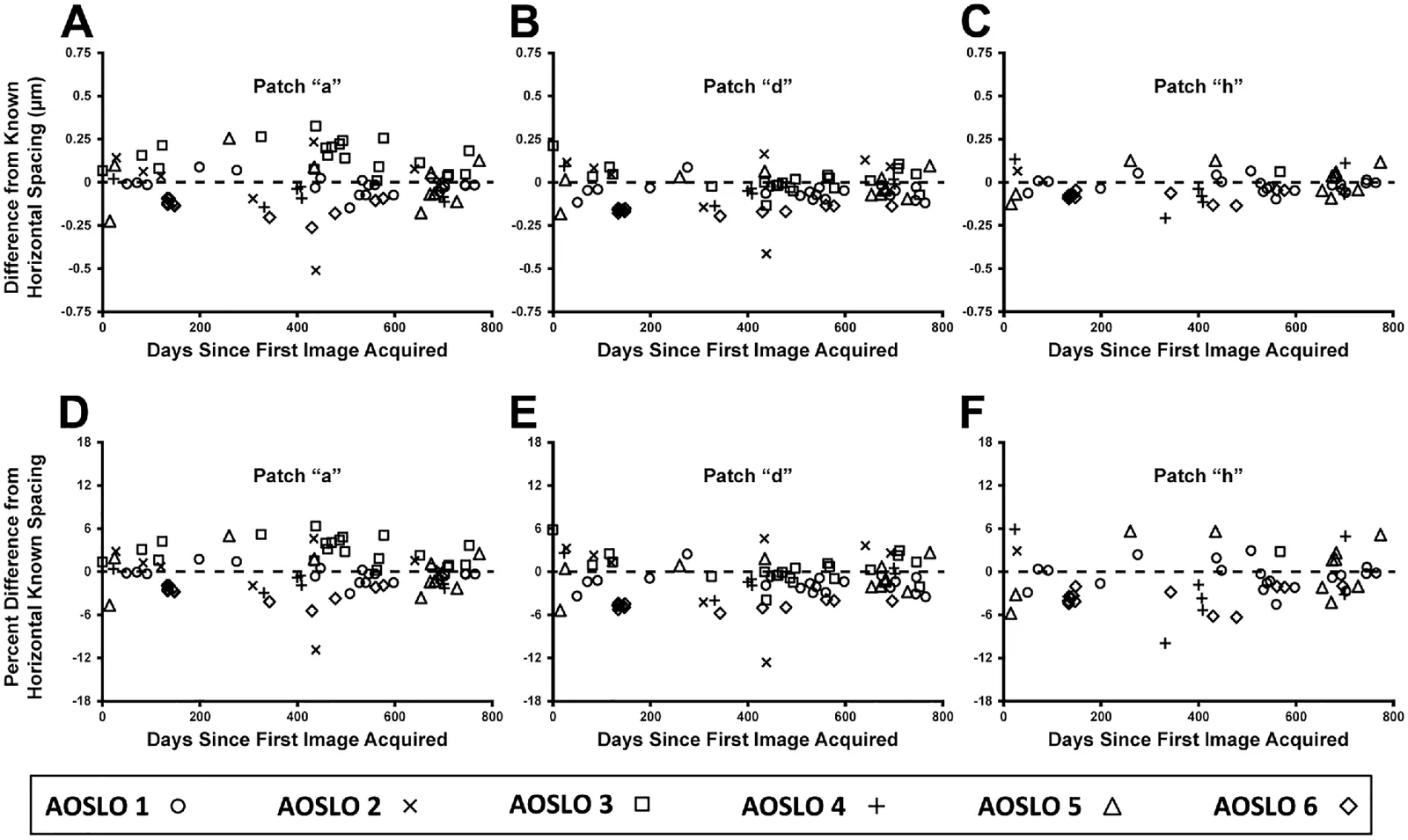
Longitudinal monitoring of horizontal “cone” spacing by patch. Displayed are the longitudinal trends of three exemplar patches of the retinal phantom (patches “a,” “d,” and “h,” as identified in Figure 1C). (**A–C**) The known horizontal “cone” spacing is subtracted from the measured horizontal “cone” spacing for each patch. Positive values indicate an overestimate, or a measured horizontal “cone” spacing greater than the known horizontal “cone” spacing, and negative values indicate an underestimate, or a measured horizontal “cone” spacing less than the known horizontal “cone” spacing. (**D–F**) The percent difference of the measured horizontal “cone” spacing from the known horizontal “cone” spacing was also calculated for each patch. Each data point represents a single AOSLO imaging session, during which the retinal phantom was imaged for longitudinal monitoring. The *dashed lines* represent a difference of zero microns or zero percent. The AOSLO used for each session is indicated by the symbol shown in the legend. The longitudinal monitoring of vertical “cone” spacing in these same exemplar patches is presented in Supplementary Figure S6. The same horizontal spacing data plotted by AOSLO rather than by patch is presented in Supplementary Figure S4, and the horizontal spacing for the remaining five phantom patches not displayed here is presented in Supplementary Figure S8.

**Figure 4.**
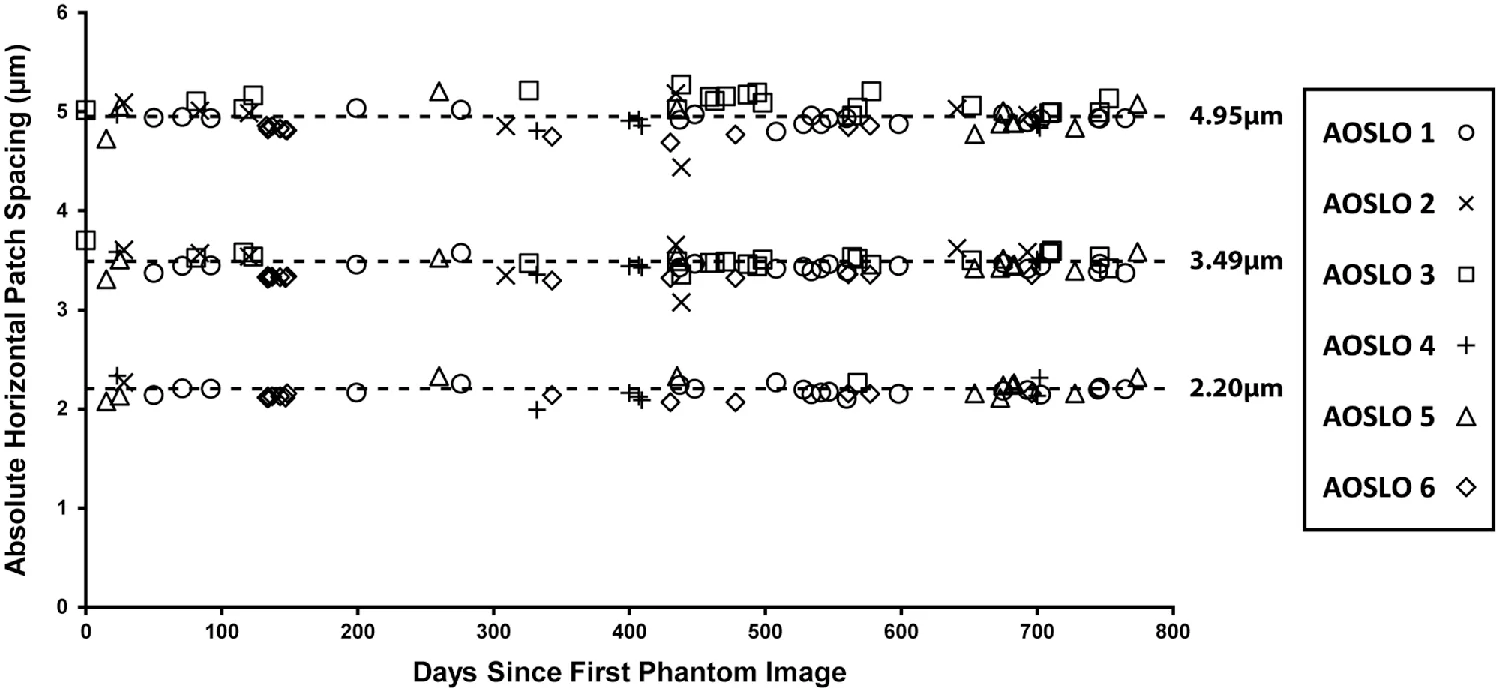
Longitudinal monitoring of horizontal “cone” spacing for three exemplar patches. The *dashed lines* represent the known horizontal spacing for patches “a” (4.95 µm), “d” (3.49 µm), and “h” (2.20 µm) as identified in Figure 1D and Table 2. The AOSLO for each session is indicated by a different symbol. Longitudinal monitoring of vertical “cone” spacing in these same exemplar patches is presented in Supplementary Figure S7.

Longitudinal monitoring over nearly 2 years revealed overall stability in spacing measurements for each of six AOSLOs monitored. Four of the AOSLOs (1, 2, 4, and 5) had horizontal “cone” spacing measurements that appeared to be very similar to, but varying about, the known horizontal “cone” spacing values over time (Supplementary Fig. S4). AOSLO 3 appeared to mostly overestimate the patch spacings on the retinal phantom (i.e., spacing measurements greater than the known spacing values), and AOSLO 6 appeared to consistently underestimate the patch spacings on the retinal phantom (i.e., spacing measurements less than the known spacing values). Similar trends were seen for vertical “cone” spacing measures (Supplementary Fig. S5). While eight patches on the retinal phantom were analyzed, we selected three representative patches to illustrate these trends (Fig. 3). The longitudinal trends of “cone” spacing measures for patches “a,” “d,” and “h” are shown as a difference from the known spacing (Figs. 3A–C) or as the percent difference from the known spacing (Figs. 3D–F). Similar trends were observed for vertical “cone” spacing measurements (Supplementary Fig. S6). The same three patches are shown in Figure 4 and Supplementary Figure S7 by their measured, or absolute, horizontal and vertical “cone” spacing, respectively. The known difference and percent difference plots for the other five patches on the retinal phantom are plotted in Supplementary Figures S8 and S9 for horizontal and vertical “cone” spacing, respectively. Each AOSLO can be identified by its unique symbol across the above-mentioned figures.

Following a series of 48 simple linear regression analyses fitted separately for the eight patches on the retinal phantom for each of the six AOSLOs, only 8 of the 48 slopes were significantly different from zero, indicating a significant change in “cone” spacing by day (Table 4). All but one of these significant slopes were negative, indicating a decrease in “cone” spacing over time. These differences were rather small, on the order of a −2.73% change in “cone” spacing per year on average. Three of the AOSLOs (2, 4, 5) had no significant slopes across the eight patches on the retinal phantom (*P* values between 0.1179 and 0.9872). Similar simple linear regression results were found for vertical “cone” spacing (Supplementary Table S3).

**Table 4.** Daily Change in Horizontal “Cone” Spacing during Longitudinal Retinal Phantom Imaging

	AOSLO 1		AOSLO 2		AOSLO 3		AOSLO 4		AOSLO 5		AOSLO 6	
Patch	Slope^a^	*P* Value	Slope^a^	*P* Value	Slope^a^	*P* Value	Slope^a^	*P* Value	Slope^a^	*P* Value	Slope^a^	*P* Value
a	−0.73	0.1671	−1.45	0.7008	−0.66	0.4770	−1.43	0.1604	−0.59	0.7335	−0.25	0.7670
b	−0.28	0.5124	−1.29	0.6944	−0.18	0.8575	−0.02	0.9792	−1.44	0.4027	−0.80	0.1179
c	−0.78	0.1578	−1.19	0.7188	−0.20	0.7481	−1.15	0.3822	−0.86	0.4959	−0.36	0.2393
d	−0.42	0.3689	−0.37	0.9097	−1.12	0.1254	−0.59	0.6787	−0.54	0.5758	−0.35	0.1629
e	−**0.96**	**0.0032**	−2.64	0.2840	−0.61	0.3117	2.04	0.4088	−0.68	0.6189	−**1.11**	**0.0194**
f	−**0.50**	**0.0472**	−0.07	0.9872	−**2.12**	**0.0009**	−3.83	0.1847	−0.69	0.5244	−**0.96**	**0.0295**
g	−**1.09**	**0.0053**	−0.11	0.9705	−0.52	0.5966	−0.80	0.3679	−0.25	0.8401	**1.32**	**0.0150**
h	−0.15	0.7221	NA	NA	NA	NA	−0.57	0.8182	−0.94	0.3824	−0.26	0.5562

a Slope = daily change in micrometers (*10^−4^). Bold = significant *P* value. NA, could not be analyzed due to lack of data points.

## Discussion

Here we demonstrate the use of a model eye-mounted retinal phantom to facilitate AOSLO imaging in a multicenter clinical trial. Our results suggest that AOSLO imaging of retinal phantoms is highly repeatable, although there can be significant differences in “cone” spacing across different AOSLOs. “Cone” spacing measurements across four unique AOSLOs varied about the known spacing values when a single retinal phantom was imaged on each AOSLO at a single time point. However, longitudinal measurements of six replicate model eye-mounted retinal phantoms, each imaged on one of six AOSLOs being used in the NAC Attack phase 3 clinical trial,^34^^,^^35^ were generally stable over 18 to 24 months.

Despite this longitudinal stability, there appears to be an offset in scale between some of the AOSLOs in this study. For example, AOSLO 3 appears to consistently overestimate the “cone” spacing of the eight patches on the retinal phantom, and AOSLO 6 appears to consistently underestimate “cone” spacing (Supplementary Figs. S4–S5), although the over- or underestimation is much less than the diameter of most cone photoreceptors. Further, two previous studies have shown that differences in cone metrics exist between AOSLOs even when they are of nearly identical optical design, with one study suggesting a difference between 2.5% and 6.9% and the other finding differences within 3%.^57^^,^^58^ Given the lack of widespread significant longitudinal changes for any one AOSLO, the retinal phantoms may aid in the development of a scaling correction factor that can be applied to cone mosaic metrics across AOSLOs for which the scale may be different (even if seemingly small). Such a correction factor could facilitate more accurate or reliable combination of data acquired on unique AOSLOs at different study sites.

While the overall stability of “cone” spacing measurements with the model eye-mounted retinal phantoms was strong, a few system attributes are known to vary between AOSLOs or even between imaging sessions. Indeed, the very nature of adaptive optics systems, and the characteristic that complicates performance standardization, is that the primary optical element (a deformable mirror [DM]) is constantly changing in response to dynamic changes in an individual's ocular aberrations. The finding that three of the four patches found to vary significantly in “cone” spacing between imaging sessions (Experiment 1) were at the field edge may be due to field curvature effects on the DM wavefront correction given the short focal length of the phantom model eye. However, it is more likely due to model eye aberrations or field-dependent system aberrations (i.e., lower order like astigmatism, coma, etc., or higher order) during imaging. A similar finding was also noted by Agrawal et al.^44^ in their analysis of similar retinal phantoms.

Variability in the following system attributes directly impacts image signal to noise and, therefore, the ability to image the phantom repeatably and reliably: (1) imaging wavelength varied across the six study sites imaging patients longitudinally for the NAC Attack clinical trial (although each used a near-infrared light source between 790 and 840 nm for imaging); (2) airy disk diameter may vary between AOSLOs and imaging session, often controlled by pinholes placed in the system prior to the system's confocal detection channel^59^; (3) image focus is adjusted by the system DM each time the retinal phantom is imaged based on the system's alignment and calibration at the time, and the “best” focus is subjective to the technician imaging the phantom; and (4) although all personnel acquiring images for the NAC Attack study were trained, certified, and adhered to the standard AOSLO imaging protocol, most sites in the longitudinal study had multiple technicians imaging the phantom at different time points, which may have introduced additional operator variability during acquisition (e.g., alignment of the model eye with the AOSLO exit pupil). Many of these “real-world” variabilities (i.e., system focus, operator variability) also exist when imaging patients enrolled in the clinical trial, and separating out differences due to the operator or measurement errors from true system drift would benefit from more objective measurement of the phantom images, as well as additional documentation of phantom placement during acquisition. Even so, until AOSLOs with well-characterized and reproducible performance specifications become widely available, our data underscore the importance of monitoring system performance throughout a longitudinal trial.

Variation in retinal phantom fabrication may introduce inherent differences between replicate retinal phantoms, which could explain the small patch-specific effects observed in some of our analyses. In their publication of the design and fabrication of retinal phantoms like those used in this study, Agrawal et al.^44^ characterize similar variation in the differences between the measured and design “cone” spacing that we saw across some of the patches analyzed in this study. Subtle variations in the 3D printing of the phantoms could also induce variability in the shape and reflectivity of the individual “cone”-like features of the patches, for which the degree of variability could be specific to the design spacing of the array of “cones” in a given patch on the retinal phantom. During longitudinal imaging of a retinal phantom, there may also be degradation, such as the gold coating affecting the polymer, which has not yet been characterized directly and could be conflated with true system drift over time.

Further variations could arise from mounting the retinal phantom substrates in the model eyes. The mounting and focusing of the model eyes were done manually for each phantom, a process that may have resulted in differences in model eye posterior chamber depth that would in turn slightly alter the magnification of each model eye-mounted retinal phantom. Despite the potential for minute magnification differences across the model eyes, we do not expect there to be changes in their retinal magnifications over time. Routine monitoring of system performance is still critical, as a change in magnification of the retinal phantom images would likely reveal a change in AOSLO system magnification. In AOSLO retinal images, an incorrect retinal magnification could lead to incorrect conclusions of how metrics like cone density and cone spacing are changing over time.^60^^,^^61^

Our study had additional limitations. First, all spacing measurements were made manually by a single unmasked observer (JK). We do not see this as a major limitation, as the agreement of repeated measurements of the same image indicates that this observer was highly repeatable (Supplementary Material S2), and minimal variance was found to be due to the grading of the image alone. Second, the method for assessing the retinal phantoms’ patches utilized only a single row of “cones” to derive their spacing. Therefore, the analyses rely on the observer's ability to assess a single row or column of “cones” in each patch rather than a more objective method where pixelwise information from the entire patch (intensities from within and between the “cones”) could be used to derive an average spacing of all the “cones” in each patch. If phantoms like this continue to be used in the future, automated analysis of phantom images would be highly beneficial. In fact, the manually graded images from the current study (there are 132 of them, 52 of which were measured twice) could be used to train an artificial intelligence/machine learning (ML) algorithm. Lastly, most images assessed in this study (Supplementary Material S1) show rotation that cannot be corrected by simple x- or y-axis translation, highlighting a limitation of how the phantoms are mounted within the model eyes. Such rotation may affect the spacing measurement accuracy because pixel-wise measurements are taken at an angle differing from zero relative to each image's x-axis.

## Conclusions

The data reported here suggest that a model eye-mounted retinal phantom is valuable for monitoring AOSLO system performance and data acquisition longitudinally. Despite overall stability of “cone” spacing measurements, variable optical design across sites produced inconsistent orientation of the phantom when imaged. The continued development of commercial AOSLOs may prove beneficial in future trials that use AOSLO-derived outcome measures, as their use would address this variability currently observed across bespoke AOSLOs. Additionally, multicenter studies that use commercially available AOSLOs^62^^,^^63^ may also benefit from the approach presented here with a phantom model eye as an important aspect of site certification for multisite or longitudinal clinical trials (Fig. 1C). Finally, the longitudinal imaging of a retinal phantom alongside study enrollees enhances the ability to discern true changes in a patient's cone mosaic from those that may be due to variations in system calibration or operator handling. Future efforts could focus on standardization of phantom fabrication and development of objective tools for analysis of the phantom images, which could help lower the barrier to entry for AO imaging-based outcome measures in future clinical trials.

## Supplementary Material

Supplement 1

Supplement 2

Supplement 3
