# Supplementary material for: Use of a Retinal Phantom to Facilitate Adaptive Optics Scanning Light Ophthalmoscopy Imaging in a Multicenter Clinical Trial
Source: Transl Vis Sci Technol. 2026 Aug 24;15(8):20. doi: 10.1167/tvst.15.8.20 (PMC13543627; doi:10.1167/tvst.15.8.20)
Supplement: Supplementary file 1 [file tvst-15-8-20_s001.pdf]

Supplementary Figures & Tables

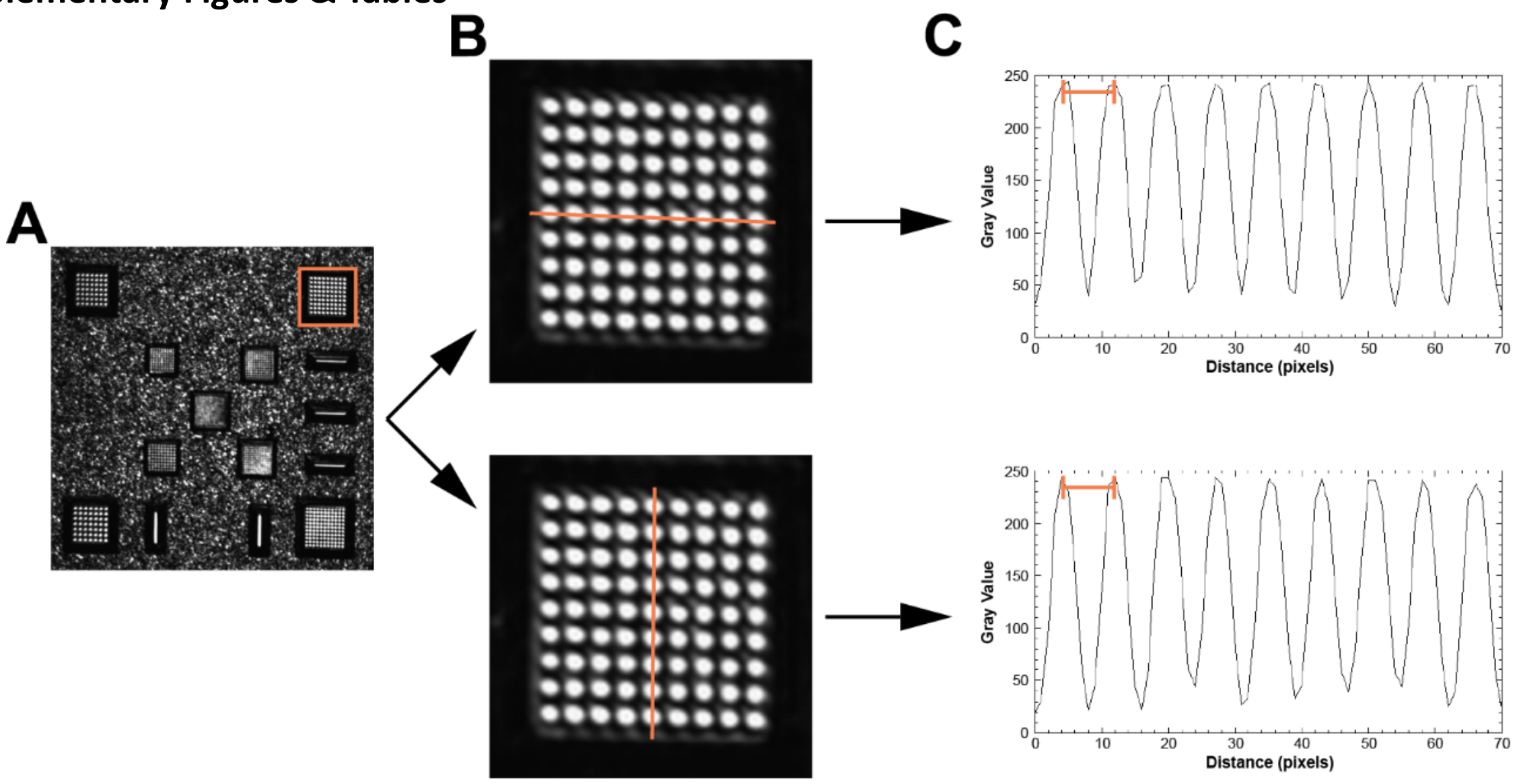

Supplementary Figure S1. Measuring "cone" spacing with a retinal phantom. (**A**) The retinal phantoms were imaged with each of eight AOSLOs to visualize the "cone-like" structures in each patch as cone photoreceptors would be imaged in the human eye. (**B**) The Line Tool in ImageJ was used to draw a line through a central row or column of "cones" in each measured patch to assess the horizontal or vertical "cone" spacing, respectively. (**C**) A pixel intensity plot profile was generated from the drawn line, and the peak-to-peak distance was measured on the profile using the Line Tool as an estimate of the "cone" spacing in pixels.

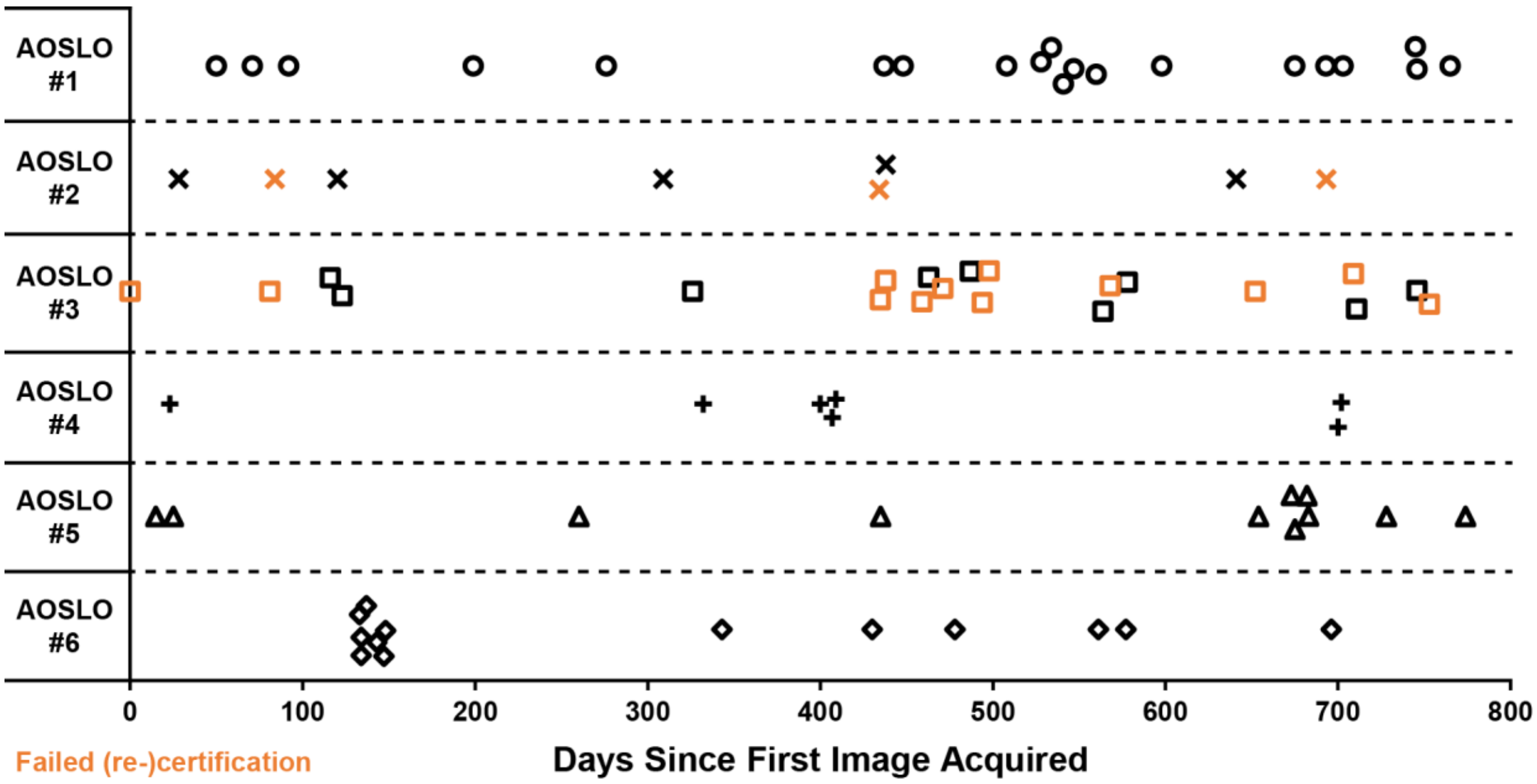

Supplementary Figure S2. Longitudinal imaging of the model eye-mounted retinal phantoms at each of the six sites enrolling patients into the AOSLO imaging substudy of the NAC Attack phase 3 clinical trial (NCT05537220). Data points are grouped laterally by each retinal phantom. AOSLO 1 was used to image that site's phantom 20 times over 715 days, AOSLO 2 was used to image that site's phantom eight times over 665 days, AOSLO 3 was used to image that site's phantom 21 times over 753 days, AOSLO 4 was used to image that site's phantom seven times over 679 days, AOSLO 5 was used to image that site's phantom 11 times over 759 days, and AOSLO 6 was used to image that site's phantom 13 times over 563 days. The orange data points are imaging dates from which the phantom image did not meet AOSLO (re-)certification guidelines for the NAC Attack Study but were still included in analyses for this study. The x-axis represents the number of days since the first image of a retinal phantom was acquired at a study site (on AOSLO 3); Day 0 is January 9th, 2023.

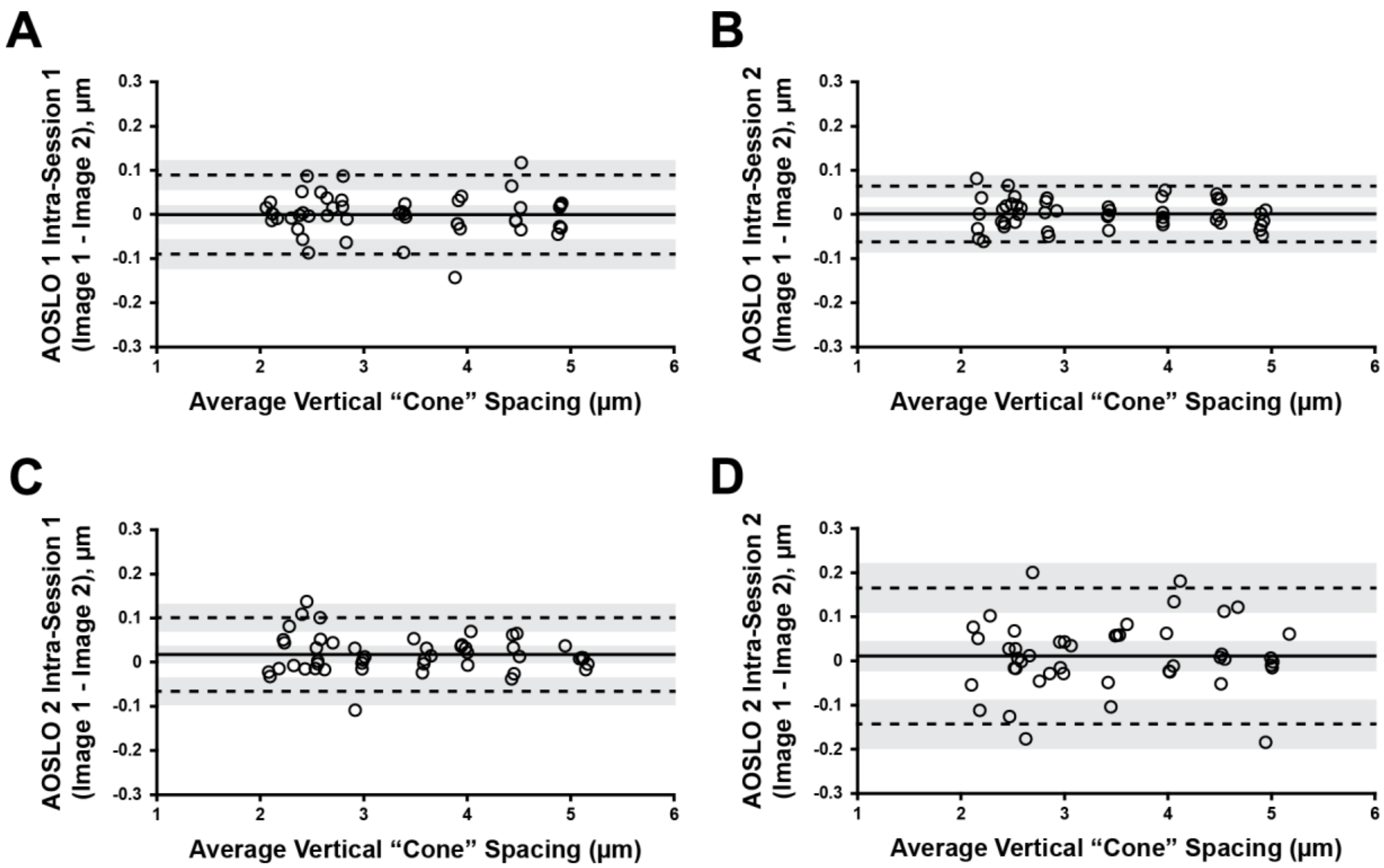

Supplementary Figure S3. Intra-session agreement of vertical "cone" spacing for six retinal phantoms. (**A**) We observed strong agreement of vertical "cone" spacing between two sets of images acquired in a single session on AOSLO 1. The *solid line* represents the mean bias of -0.0003 µm, and the *dashed lines* represent the upper (0.089 µm) and lower (-0.090 µm) limits of agreement. (**B**) Strong agreement was also observed for vertical "cone" spacing between two sets of images acquired during a separate imaging session on AOSLO 1. The *solid line* represents the mean bias of 0.0009 µm, and the *dashed lines* represent the upper (0.064 µm) and lower (-0.062 µm) limits of agreement. (**C**) A very small bias was observed on AOSLO 2 for vertical "cone" spacing between repeated imaging within the first session (*i.e.*, the 99% CIs of the mean did not include zero). The *solid line* represents the mean bias of 0.017 µm (CIs: 0.0009 µm, 0.034 µm), and the *dashed lines* represent the upper (0.101 µm) and lower (-0.066 µm) limits of agreement. (**D**) We did observe strong agreement, however, of vertical "cone" spacing between repeated imaging in a second session on another day on AOSLO 2. The *solid line* represents the mean bias of 0.011 µm, and the *dashed lines* represent the upper (0.165 µm) and lower (-0.143 µm) limits of agreement. The *gray shading* represents the 99% CIs about the mean bias and the limits of agreement.[64] Each vertical grouping of data points indicates a patch on the phantom with patch "a" being the greatest spacing on the right and patch "h" being the smallest spacing on the left.

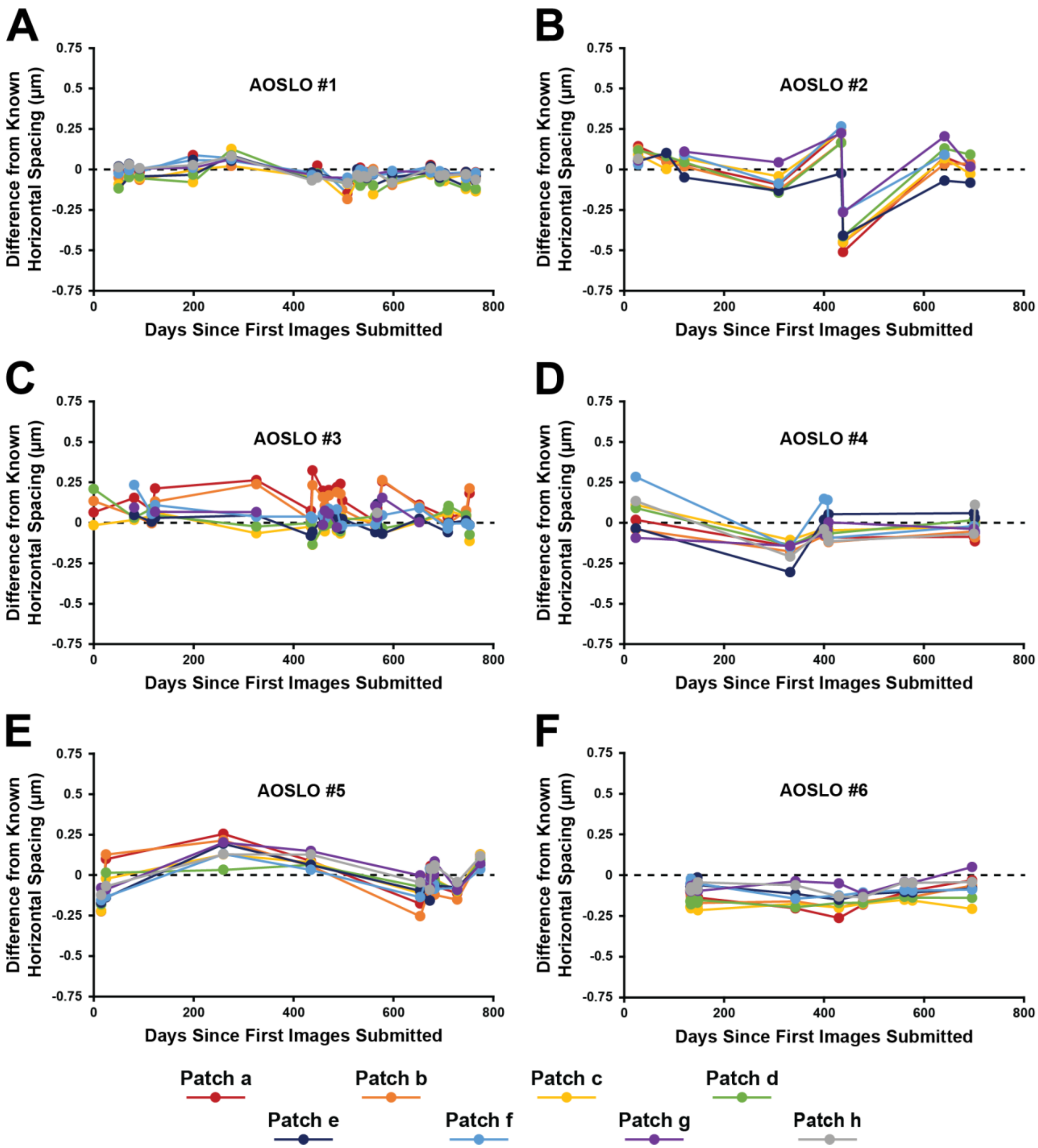

Supplementary Figure S4. Longitudinal monitoring of horizontal "cone" spacing by phantom. (**A-F**) The known horizontal "cone" spacing is subtracted from the measured horizontal "cone" spacing for the eight patches on the retinal phantom each time it was imaged with AOSLO 1, 2, 3, 4, 5, and 6, respectively. Positive values indicate an overestimate, or a measured horizontal "cone" spacing greater than the known horizontal "cone" spacing, and negative values indicate an underestimate, or a measured horizontal "cone" spacing less than the known horizontal "cone" spacing. Longitudinal monitoring of vertical "cone" spacing by phantom is presented in **Supplementary Figure S5**.

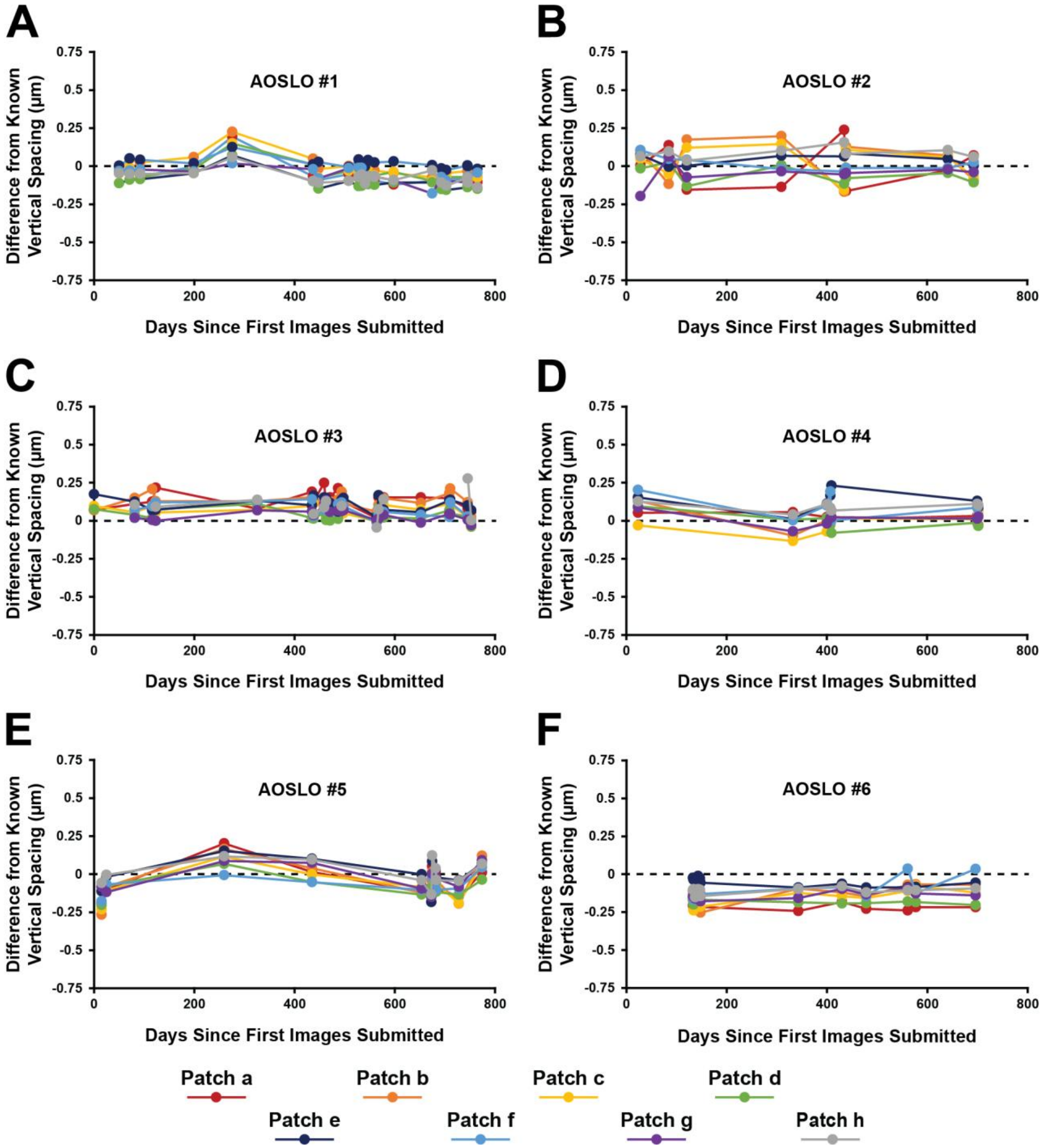

Supplementary Figure S5. Longitudinal monitoring of vertical "cone" spacing by phantom. (**A-F**) The known vertical "cone" spacing is subtracted from the measured vertical "cone" spacing for the eight patches on the retinal phantom each time it was imaged with AOSLO 1, 2, 3, 4, 5, and 6, respectively. Positive values indicate an overestimate, or a measured vertical "cone" spacing greater than the known vertical "cone" spacing, and negative values indicate an underestimate, or a measured vertical "cone" spacing less than the known vertical "cone" spacing.

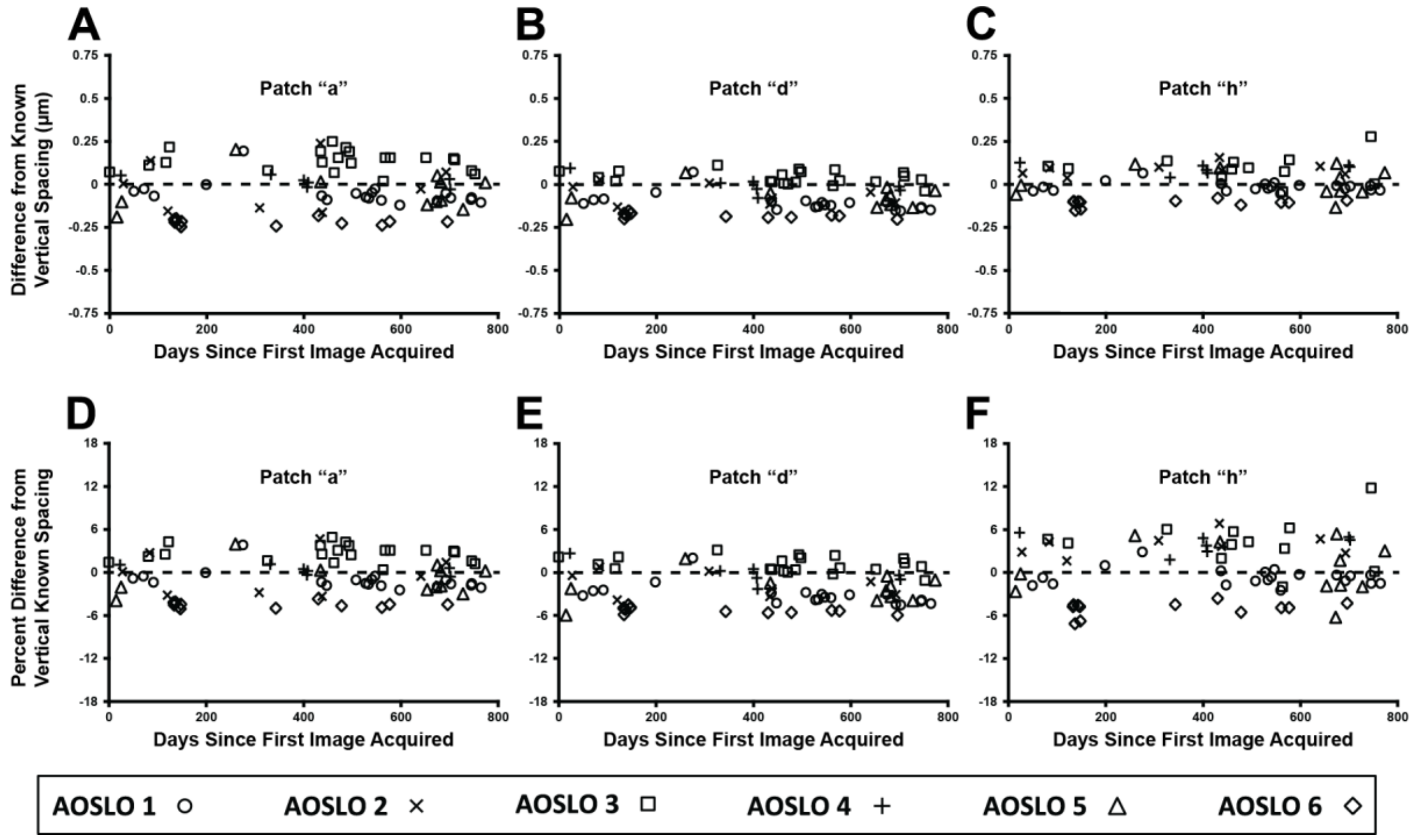

Supplementary Figure S6. Longitudinal monitoring of vertical "cone" spacing by patch. Displayed are the longitudinal trends of three exemplar patches of the retinal phantom (patches "a", "d", and "h" as identified in **Figure 1D**). (**A-C**) The known vertical "cone" spacing is subtracted from the measured vertical "cone" spacing for each patch. Positive values indicate an overestimate, or a measured vertical "cone" spacing greater than the known vertical "cone" spacing, and negative values indicate an underestimate, or a measured vertical "cone" spacing less than the known vertical "cone" spacing. (**D-F**) The percent difference of the measured vertical "cone" spacing from the known vertical "cone" spacing was also calculated for each patch. Each data point represents a single AOSLO imaging session during which the retinal phantom was imaged for longitudinal monitoring. The *dashed lines* represent a difference of zero µm or zero percent. The AOSLO used for each session is indicated by the symbol shown in the legend. The vertical spacing for the remaining five phantom patches not displayed here is presented in **Supplementary Figure S9**.

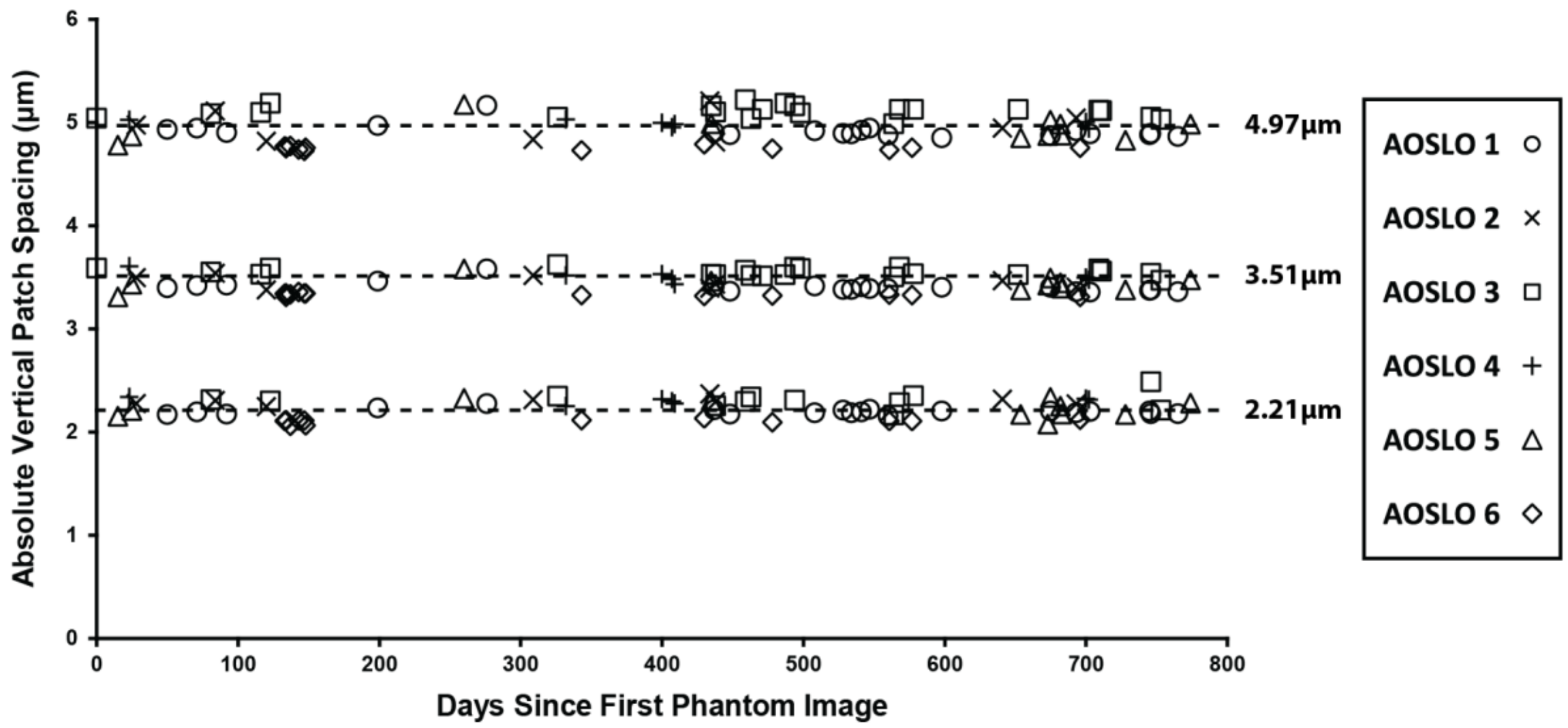

Supplementary Figure S7. Longitudinal monitoring of vertical "cone" spacing for three exemplar patches. The *dashed lines* represent the known vertical spacing for patches "a" (4.97 µm), "d" (3.51 µm), and "h" (2.21 µm) as identified in **Figure 1D** and **Supplementary Table S2**. The AOSLO for each session is indicated by a different symbol.

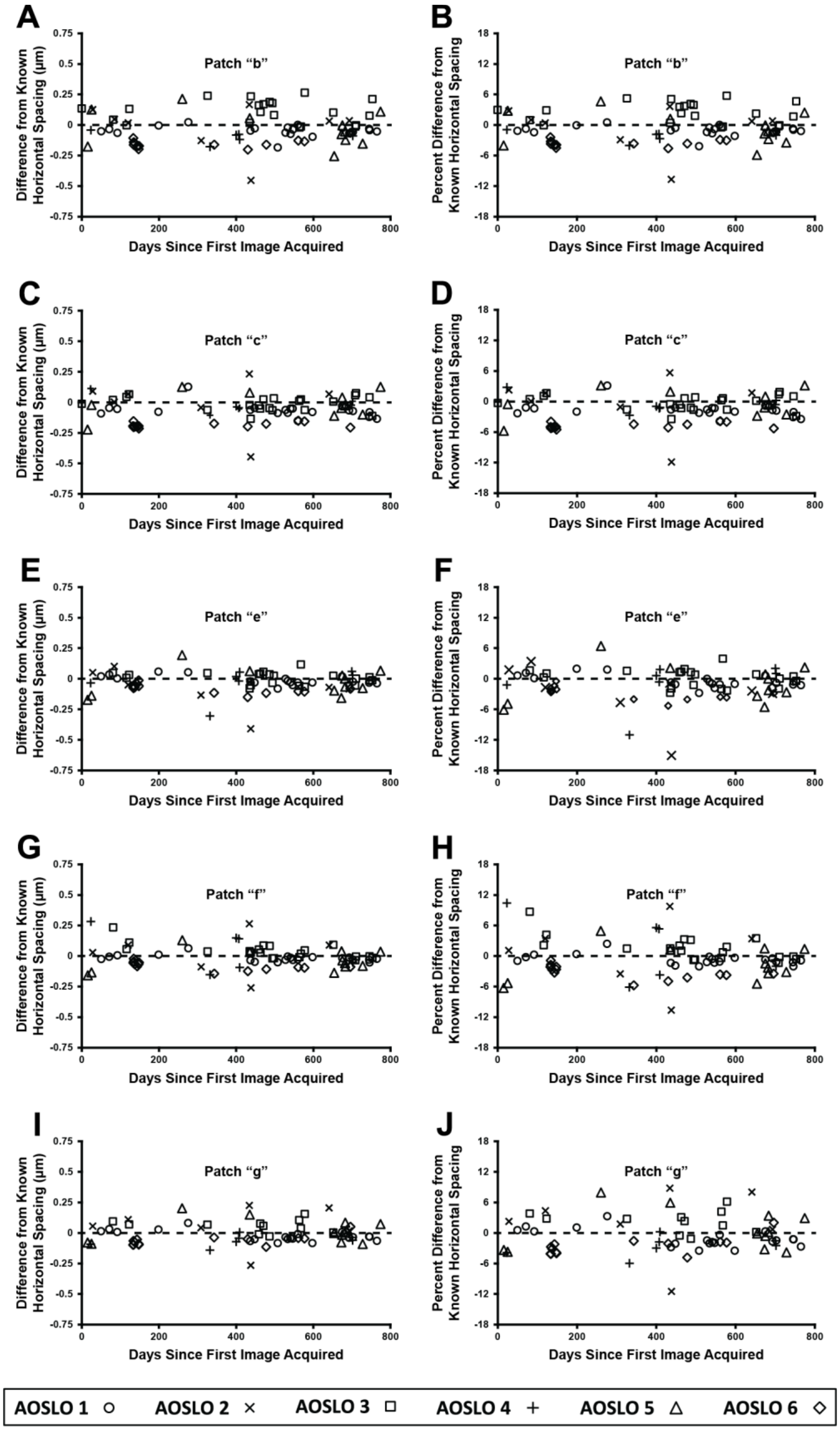

Supplementary Figure S8. Longitudinal monitoring of horizontal "cone" spacing by patch. Displayed are the longitudinal trends of the five patches of the retinal phantom not displayed in **Figure 3** (patches "b",

"c", "e", "f", and "g" as identified in **Figure 1D**). (**A, C, E, G, I**) The known horizontal "cone" spacing is subtracted from the measured horizontal "cone" spacing for each patch. Positive values indicate an overestimate, or a measured horizontal "cone" spacing greater than the known horizontal "cone" spacing, and negative values indicate an underestimate, or a measured horizontal "cone" spacing less than the known horizontal "cone" spacing. (**B, D, F, H, J**) The percent difference of the measured horizontal "cone" spacing from the known horizontal "cone" spacing was also calculated for each patch. Each data point represents a single AOSLO imaging session during which the retinal phantom was imaged for longitudinal monitoring. The *dashed lines* represent a difference of zero µm or zero percent. Each session during which a retinal phantom was imaged is indicated by a symbol according to which AOSLO was used for imaging. Longitudinal monitoring of vertical "cone" spacing by patch is presented in **Supplementary Figures S6** and **S9**.

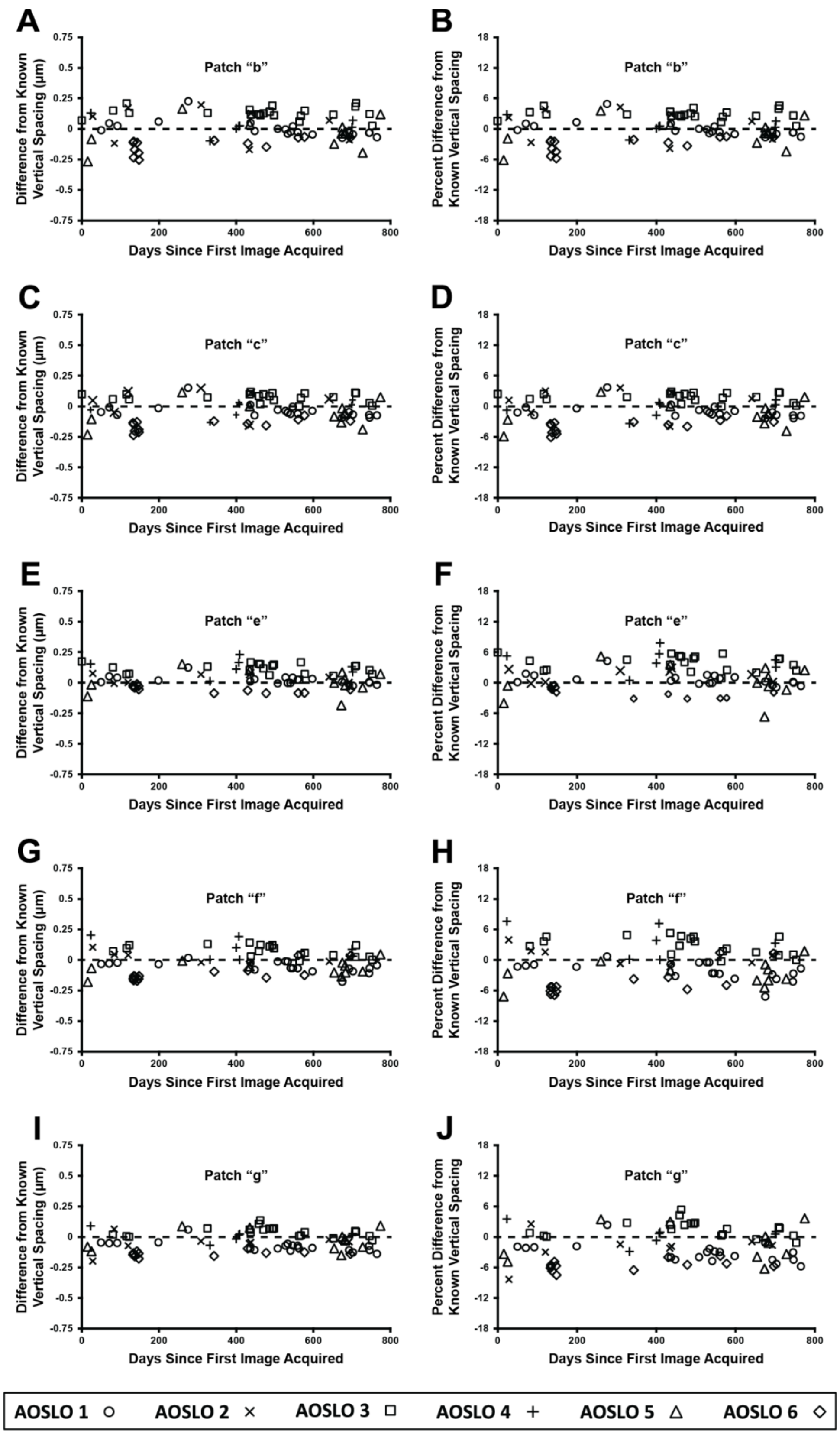

Supplementary Figure S9. Longitudinal monitoring of vertical "cone" spacing by patch. Displayed are the longitudinal trends of the five patches of the retinal phantom not displayed in **Supplementary Figure S6**

(patches “b”, “c”, “e”, “f”, and “g” as identified in **Figure 1D**). (**A, C, E, G, I**) The known vertical “cone” spacing is subtracted from the measured vertical “cone” spacing for each patch. Positive values indicate an overestimate, or a measured vertical “cone” spacing greater than the known vertical “cone” spacing, and negative values indicate an underestimate, or a measured vertical “cone” spacing less than the known vertical “cone” spacing. (**B, D, F, H, J**) The percent difference of the measured vertical “cone” spacing from the known vertical “cone” spacing was also calculated for each patch. Each data point represents a single AOSLO imaging session during which the retinal phantom was imaged for longitudinal monitoring. The *dashed lines* represent a difference of zero µm or zero percent. Each session during which a retinal phantom was imaged is indicated by a symbol according to which AOSLO was used for imaging.

Supplementary Table S1.
Examining Differences in Vertical "Cone" Spacing Measurements Between Phantoms

	Retinal Phantom 1		Retinal Phantom 2		Retinal Phantom 3		Retinal Phantom 4		Retinal Phantom 5		Retinal Phantom 6	
**Patch**	**Effect Size[a]**	***P* Value**	**Effect Size[a]**	***P* Value**	**Effect Size[a]**	***P* Value**	**Effect Size[a]**	***P* Value**	**Effect Size[a]**	***P* Value**	**Effect Size[a]**	***P* Value**
**a**	-0.0179	0.3034	-0.0131	0.4549	0.0133	0.4461	0.0109	0.5306	0.0001	0.9929	0.0066	0.7067
**b**	0.0100	0.4899	0.0222	0.1226	**0.0458**	**0.0011**	**-0.0373**	**0.0086**	-0.0163	0.2584	-0.0244	0.0893
**c**	0.0092	0.4530	**-0.0330**	**0.0063**	**0.0258**	**0.0344**	-0.0053	0.6700	-0.0116	0.3462	0.0149	0.2277
**d**	-0.0106	0.5062	0.0022	0.8887	-0.0004	0.9813	-0.0152	0.3361	**0.0330**	**0.0354**	-0.0091	0.5670
**e**	0.0241	0.1277	**0.0450**	**0.0039**	0.0155	0.3292	-0.0084	0.5979	0.0100	0.5298	**-0.0863**	**0.0000**
**f**	0.0324	0.1495	-0.0248	0.2707	**0.1014**	**0.0000**	-0.0228	0.3116	-0.0175	0.4359	**-0.0686**	**0.0018**
**g**	0.0164	0.3712	**0.0433**	**0.0171**	0.0274	0.1344	-0.0335	0.0669	**-0.0577**	**0.0013**	0.0041	0.8249
**h**	0.0016	0.9372	-0.0272	0.1802	0.0121	0.5748	**0.0544**	**0.0065**	0.0167	0.4144	**-0.0563**	**0.0049**

[a]Effect size provided in micrometers. Effect size = estimate of "cone" spacing difference between one phantom and an average of the other five. Bold = significant *P* value.

Supplementary Table S2.
Measured Versus Known Vertical "Cone" Spacing Across Four AOSLOs

	a	b	c	d	e	f	g	h
**Known Spacing[a]**	4.97	4.50	4.01	3.51	2.85	2.58	2.47	2.21
**AOSLO 1**								
**Measured spacing[a]**	4.96	4.50	3.95	3.41	2.83	2.65	2.42	2.21
**Difference (µm)**	-0.014	-0.004	-0.057	-0.102	-0.016	0.070	-0.054	0.001
**Difference (%)**	-0.29	-0.10	-1.43	-2.96	-0.56	2.68	-2.19	0.06
**AOSLO 2**								
**Measured spacing[a]**	4.99	4.48	4.01	3.54	2.93	2.76	2.54	2.14
**Difference (µm)**	0.021	-0.017	-0.001	0.032	0.077	0.179	0.073	-0.068
**Difference (%)**	0.41	-0.37	-0.04	0.90	2.68	6.72	2.93	-3.15
**AOSLO 7**								
**Measured spacing[a]**	5.05	4.44	3.89	3.51	2.93	2.59	2.44	2.25
**Difference (µm)**	0.078	-0.058	-0.118	0.000	0.075	0.013	-0.028	0.041
**Difference (%)**	1.55	-1.30	-2.98	0.01	2.61	0.50	-1.15	1.83
**AOSLO 8**								
**Measured spacing[a]**	5.06	4.55	4.08	3.57	2.95	2.60	2.52	2.34
**Difference (µm)**	0.093	0.049	0.066	0.061	0.098	0.018	0.054	0.134
**Difference (%)**	1.86	1.09	1.63	1.73	3.39	0.69	2.17	5.88
**Four-system average**								
**Measured spacing[a]**	5.01	4.49	3.98	3.51	2.91	2.65	2.48	2.24
**Difference (µm)**	0.044	-0.008	-0.027	-0.002	0.059	0.070	0.011	0.027
**Difference (%)**	0.88	-0.17	-0.70	-0.08	2.03	2.65	0.44	1.16

[a]Known and measured spacing values are in micrometers.

Supplementary Table S3.
Assessing the Impact of AOSLO on Vertical "Cone" Spacing Measurements

	AOSLO #1		AOSLO #2		AOSLO #7		AOSLO #8	
**Patch**	**Effect Size[a]**	***P* Value**	**Effect Size[a]**	***P* Value**	**Effect Size[a]**	***P* Value**	**Effect Size[a]**	***P* Value**
**a**	**-0.0784**	**0.0276**	-0.0317	0.4574	0.0445	0.2828	0.0655	0.0890
**b**	0.0041	0.9181	-0.0121	0.7592	**-0.0677**	**0.0430**	**0.0757**	**0.0151**
**c**	-0.0391	0.5522	0.0348	0.5986	**-0.1203**	**0.0268**	**0.1247**	**0.0188**
**d**	**-0.1336**	**0.0007**	0.0453	0.4455	0.0033	0.9560	0.0849	0.1215
**e**	**-0.0997**	**0.0003**	0.0248	0.5726	0.0221	0.6158	0.0528	0.2020
**f**	0.0001	0.9986	**0.1457**	**0.0007**	-0.0761	0.2208	-0.0697	0.2675
**g**	-0.0867	0.0672	0.0827	0.0856	-0.0529	0.3105	0.0569	0.2722
**h**	-0.0340	0.6349	**-0.1272**	**0.0329**	0.0185	0.7973	**0.1427**	**0.0093**

[a]Effect size provided in micrometers. Effect size = estimate of "cone" spacing difference between one AOSLO and an average of the other three. Bold = significant *P* value.

Supplementary Table S4.
Daily Change in Vertical "Cone" Spacing During Longitudinal Retinal Phantom Imaging

	AOSLO #1		AOSLO #2		AOSLO #3		AOSLO #4		AOSLO #5		AOSLO #6	
**Patch**	**Slope[a]**	***P* Value**	**Slope[a]**	***P* Value**	**Slope[a]**	***P* Value**	**Slope[a]**	***P* Value**	**Slope[a]**	***P* Value**	**Slope[a]**	***P* Value**
**a**	**-1.28**	**0.0464**	0.57	0.8185	-0.14	0.8234	-0.81	0.1734	0.30	0.8256	-0.04	0.8862
**b**	**-1.63**	**0.0076**	-1.39	0.5544	-0.16	0.7366	-0.63	0.6474	1.07	0.4795	**1.89**	**0.0189**
**c**	-0.94	0.0741	-1.02	0.5673	-0.30	0.4266	1.32	0.2845	0.73	0.5608	**1.43**	**0.0158**
**d**	**-1.16**	**0.0150**	-0.93	0.3339	-0.54	0.1659	-1.56	0.0985	0.22	0.8001	**-0.41**	**0.0241**
**e**	**-0.73**	**0.0224**	0.05	0.9344	-0.30	0.5036	-0.37	0.7880	0.17	0.8877	**-0.94**	**0.0040**
**f**	**-1.04**	**0.0132**	-1.17	0.0845	**-1.10**	**0.0381**	-1.71	0.2817	0.65	0.3843	**2.50**	**0.0024**
**g**	**-1.03**	**0.0130**	0.75	0.5293	-0.16	0.7396	-0.56	0.5509	0.38	0.7143	0.51	0.1251
**h**	-0.16	0.5446	0.40	0.5062	0.15	0.9062	-0.02	0.9735	-0.06	0.9531	0.30	0.3001

[a]Slope = daily change in micrometers (*10^{-4}). Bold = significant *P* value.
