# Supplementary material for: Use of a Retinal Phantom to Facilitate Adaptive Optics Scanning Light Ophthalmoscopy Imaging in a Multicenter Clinical Trial
Source: Transl Vis Sci Technol. 2026 Aug 24;15(8):20. doi: 10.1167/tvst.15.8.20 (PMC13543627; doi:10.1167/tvst.15.8.20)
Supplement: Supplementary file 2 [file tvst-15-8-20_s002.zip › Suppl Material S1 - All Phantom Images/Supplementary Material S1.pdf]

Supplementary Material S1. Images of the model eye-mounted retinal phantoms included in analyses. All images included in the analyses have been organized by experiment and made available to the reader. This page explains the file naming convention for the images.

Image File Naming Convention:
[AOSLO #]_[Phantom #]_[Imaging Date]_[im #]_[Horizontal and Vertical Image Scale].tif
Example: AOSLO3_Phantom1_20230109_H_0p626_V_0p621.tif

[AOSLO #]: numerical identifier for which of the eight AOSLO system was used to image the retinal phantom
"#" = 1, 2, 3, 4, 5, 6, 7, or 8

[Phantom #]: numerical identifier for which of the six replicate retinal phantoms was imaged
"#" = 1, 2, 3, 4, 5, or 6

[Imaging Date]: the date on which the retinal phantom was imaged
Formatted as YYYYMMDD

[im #]: the first or second image acquired of the phantom if multiple were acquired on the same day
"#" = 1 or 2

[Horizontal and Vertical Image Scale]: the image's scale in microns per pixel (from its native orientation at acquisition) in the horizontal and vertical dimension
Formatted as H_0p### and V_0p###
_H_0p###_V_0p###

Note: All images of the retinal phantoms are nominal 1x1° field of view in size and were assessed in their native orientation at acquisition. In the **Supplementary Material S1** folder, phantom images have been transformed (rotated and/or flipped) if needed to display all of the images in the same orientation. The ReadMe.txt file lists whether and how an image was transformed. For example:

a. Reference Orientation from AOSLO #1:

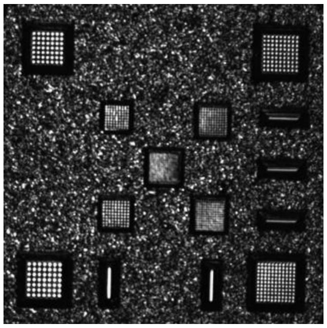

b. Example Transformation for AOSLO #5:

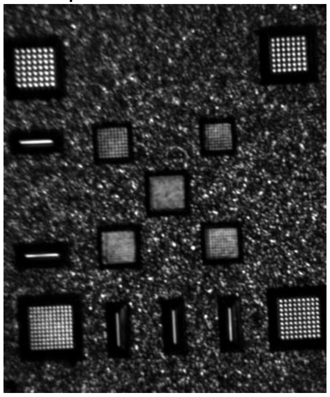

→→

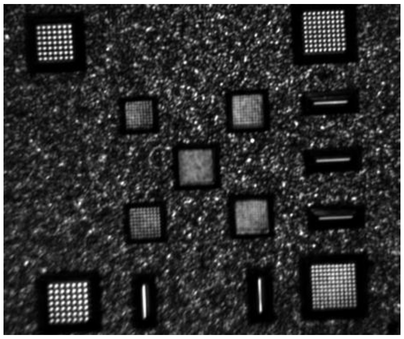

Rotated 90° to the left
